# Biocompatible Sulfobetaine Polymer‐Artemisinin Conjugates Inducing Ferroptosis in Cancer Cells: Synthesis by Mechanochemical Solid‐State Polymerization and Characterization

**DOI:** 10.1002/marc.70341

**Published:** 2026-06-22

**Authors:** Naoki Doi, Yukinori Yamauchi, Yasushi Sasai, Ryosuke Nagai, Ayaka Koike, Madoka Ikawa, Toru Yoshimoto, Satoshi Yamada, Masayuki Kuzuya, Shin‐ichi Kondo

**Affiliations:** ^1^ Laboratory of Pharmaceutical Physical Chemistry Gifu Pharmaceutical University Gifu Japan; ^2^ Department of Pharmaceutical Physical Chemistry College of Pharmaceutical Sciences Matsuyama University Matsuyama Ehime Japan; ^3^ Faculty of Pharmacy Gifu University of Medical Science Gifu Japan

**Keywords:** artemisinin, drug delivery system, ferroptosis, mechanochemical solid‐state polymerization, polymer‐drug conjugates, sulfobetaine polymer

## Abstract

Artemisinin derivatives (ARTs) induce ferrous iron‐dependent cell death (ferroptosis) by generating free radicals. Since the concentration of ferrous ions within cancer cells is high, the specific delivery of ARTs into cancer cells is advantageous for cancer therapy. Herein, we fabricate the biocompatible sulfobetaine polymer‐ARTs conjugates through the mechanochemical solid‐state copolymerization of sulfobetaine methacrylate (SBMA) and ARTs‐conjugated monomer. A polymer conversion rate of more than 90% is achieved within 60 min of copolymerization. The number average molecular weight and heterogeneity of resulting water‐soluble PSBMA‐ARTs conjugates are 8,000 g mol^−1^ and 1.10, respectively. The progressive changes in ESR spectrum intensity with DMPO as a spin trapping agent reveal that the PSBMA‐ARTs conjugates ferrous ion‐dependently produce free radicals. The PSBMA‐ARTs conjugates indicate the cytotoxic activities against human hepatoblastoma cell lines, and the IC_50_ of artesunate and PSBMA‐ARTs conjugates are 136 and 211 µm, respectively. Furthermore, it is demonstrated that PSBMA‐ARTs conjugates internalized into cells generate the free radicals and induce the lipid peroxidation in cell membranes or intracellularly involved in ferroptosis, determined by the live‐cell imaging with C11‐BODIPY. This novel drug carrier, which does not require drug release, is a promising cancer ferroptosis inducer for expanding pharmaceutical treatment strategies.

## Graphical abstract

The amphiphilic copolymer incorporating a hydrophobic chain conjugated with artesunate into a biocompatible sulfobetaine polymer is synthesized by solid‐state copolymerization of sulfobetaine methacrylate and methacrylamide derivative conjugating artesunate. It internalizes into cancer cells while avoiding protein adsorption, and induces cancer ferroptosis by generating free radicals via the reaction with ferrous iron.

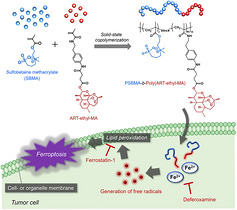

## 1 Introduction

Macromolecular drugs (e.g. polymeric prodrugs, polymer‐drug conjugates, antibodies, cell‐penetrating peptides, and oligonucleotides) have attracted attention for cancer therapy, since they may be expected to undergo passive targeting into solid tumor tissues [1, 2]. The intracellular iron concentration of most cancer cells is higher than that of normal cells, due to the enhanced expression of transferrin receptor in cancer cells [3, 4, 5, 6, 7, 8]. Therefore, iron‐responsive macromolecular drugs may be beneficial as anti‐cancer agents.

Artemisinin (ART), isolated from the Chinese plant *Artemisia annua L*., has been widely used for anti‐malarial treatment. ART and its derivatives (ARTs), such as dihydroartemisinin and artesunate, are sesquiterpene lactones with an endoperoxide bridge, which can produce free radicals via the cleavage of the endoperoxide bridge with ferrous iron [9, 10, 11, 12, 13, 14]. It has widely been reported that ARTs induce the iron‐dependent cellular death, which is known as ferroptosis. ARTs, however, have short half‐lives (less than 45 min for artesunate and 21~64 min for dihydroartemisinin) in blood and poor cancer‐selective migration [15, 16, 17, 18]. Thus, macromolecular drugs loaded with ARTs would be taken advantage of ARTs delivery into cancer cells and could induce ferroptosis [19, 20, 21, 22, 23]. In 2005, H. Lai et al. first reported that the artemisinin‐transferrin conjugates, synthesized by covalent tagging of 4~16 molecules of artemisinin into the *N*‐glycoside moieties in a holo‐transferrin, were more transported into acute T lymphoblastic leukaemia cells than normal human lymphocytes [3]. I. Nakase et al. also reported that the artemisinin‐transferrin conjugates showed cytotoxic effects against the human prostate carcinoma cells, and the cytotoxicity was strongly dependent on the number of artemisinin units per transferrin [4, 5, 6]. Additionally, M. Ebara et al. have reported on the design of a hydrophobic polymethacrylic acid derivative possessing artesunate as a repeating unit in the side chains [24]. Therefore, abundant conjugates of ARTs into water‐soluble linear polymers might be unprecedentedly promising as an ARTs carrier.

Polymer‐drug conjugates, in which drugs are attached to the side chains or terminal of a polymer, are well‐known as one of drug delivery system (DDS) carrier. Most of the polymer‐drug conjugates may be represented in the same sense as the polymeric prodrugs, which can release the drugs from the polymer. However, polymer‐drug conjugates that exhibit the pharmaceutical efficacy without releasing the drug would strictly be distinct from polymeric prodrugs, in which the drug release from the polymer is crucial. Although the polymer‐drug conjugates can easily be synthesized by the conjugation of the drugs into polymer side‐chains without considering the drug quantification, the conjugation would be inconsistent due to the steric hindrance of the polymer. Therefore, polymer‐drug conjugates, in which drugs are conjugated to polymer side chains via repeating units, may enable drug delivery into cancer cells at high concentrations. The polymerization of monomers conjugating drugs would be advantageous for the design and synthesis of polymer‐drug conjugates.

We have reported the mechanochemical solid‐state polymerization of solid monomers with vibratory ball‐milling in a nitrogen atmosphere at room temperature to synthesize functional polymers [25, 26, 27, 28, 29, 30, 31]. The mechanochemical solid‐state polymerization requires no solvents, catalysts, or initiators. The initiation reaction could be triggered by solid‐state single electron transfer (SSET) generated from the collision of the stainless steel vessel and ball [32]. The resulting high molecular weight polymers (ca. 3–5 × 10^4^ g/mol) produced in the initial stage of solid‐state polymerization at 60 Hz would undergo the main‐chain scission via the mechanical energy, and the end‐chain radicals would be generated. The polymer end‐chain radicals could also play the role as macroinitiators for the solid‐state polymerization of residual vinyl monomers, and the polymerization would successfully achieve the quantitative conversion with prolonged milling [33]. Thus, in the mechanochemical solid‐state polymerization, it may not be necessary to consider the affinity between vinyl monomers and solvents or catalysts, unlike liquid‐phase radical polymerization. The mechanochemical solid‐state polymerization also allows the copolymerization of multiple vinyl monomers. Recently, we have revealed the structural properties of the resulting block copolymers synthesized by mechanochemical solid‐state copolymerization of sulfobetaine methacrylate (SBMA) and *N*‐benzyl methacrylamide, using ^1^H/DOSY‐NMR and DSC measurements [30]. It was demonstrated that the resulting amphiphilic PSBMA could internalize into human hepatoblastoma cell lines (HepG2) within 5 min. Although the intact PSBMA cannot interact with HepG2 due to the higher biocompatibility, the amphiphilic PSBMA with a slightly hydrophobic chain would interact with HepG2 while avoiding protein adsorption. Therefore, amphiphilic PSBMA incorporating ARTs into its hydrophobic chain may contribute to an innovative cancer treatment strategy that efficiently delivers ARTs into cancer cells.

**FIGURE 1:**
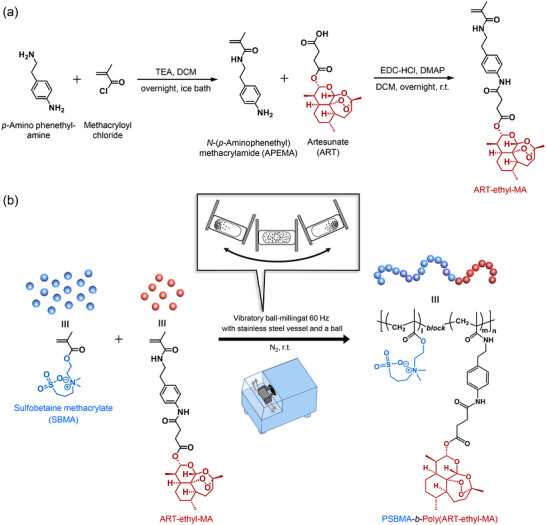
(a) Synthetic scheme of methacrylamide derivative conjugating artesunate (ART‐ethyl‐MA). (b) Schematic illustration of PSBMA‐ARTs conjugates synthesized by the solid‐state copolymerization of SBMA and ART‐ethyl‐MA.

In this study, we aimed to develop iron‐responsive polymer‐drug conjugates generating free radicals. In order to provide the polymer‐ARTs conjugate with water‐solubility, protein antifouling, and biocompatibility, PSBMA as the hydrophilic chains of the polymer‐ARTs conjugate was selected. We recently successfully synthesized a novel solid methacrylamide derivative of ARTs [31] and carried out the mechanochemical solid‐state copolymerization of ARTs‐conjugated methacrylamide and SBMA in this study, as shown in Figure 1. The resulting polymer, PSBMA‐ARTs conjugates, was characterized for the productivity of free radicals by ESR and its simulation analysis, the antifouling effects of serum proteins, and the cytotoxicity effects caused by lipid peroxidation against HepG2 were diligently examined.

## 2 Materials and Methods

### 2.1 Materials

The following reagents were special or first‐grade products and were used directly. Albumin from human serum, 2‐(4‐aminophenyl)ethylamine, ammonium iron (II) sulfate hexahydrate (Fe(NH_4_)_2_(SO_4_)_2_·6H_2_O), fibrinogen from human plasma, human recombinant insulin, iron (III) chloride hexahydrate (FeCl_3_·6H_2_O), penicillin‐streptmycin (×100) solution, triethylamine (TEA), 1,3‐propanesultone, 2‐(dimethylamino)ethyl methacrylate, selenous acid, sodium formate, and 2,2,2‐trifluoroethanol (TFE)‐*d_2_* were purchased from Fujifilm Wako Pure Chemical Co. (Osaka, Japan). Acetone, ethanol (EtOH), ethyl acetate, ethylenediaminetetraacetic acid (EDTA), maleic acid, methanol (MeOH), and *n*‐hexane were obtained from Kishida Chemical Co., Ltd (Osaka, Japan). NaCl, magnesium sulfate dehydrate (MgSO_4_), and sodium sulfate (Na_2_SO_4_) were purchased from Nacalai Tesque Inc. (Kyoto, Japan). Chloroform‐*d* (CDCl_3_), D_2_O, and dichloromethane (DCM) were obtained from Kanto Chemical Co., Inc. Artesunate, dexamethasone, 4‐dimethylaminopyridine (DMAP), 1‐(3‐dimethylaminopropyl)‐3‐ethylcarbodiimide hydrochloride (EDC‐HCl), L‐glutamine, linoleic acid, methacryloyl chloride, deferoxamine mesylate, and ferrostatin‐1 were obtained from Tokyo Chemical Industry Co., Ltd, (Tokyo, Japan). Methacryloyl chloride was distilled before use. Human transferrin was obtained from Sigma‐Aldrich. Reagents other than those listed above were also used directly. The Spectra/Por 3 or 7 Dialysis Membrane Standard RC Tubing (molecular weight cut‐off (MWCO), 3,500 or 1,000) was purchased from Spectrum Laboratories, Inc. Milli‐Q water (Millipore, 18.2 MΩ cm at 25°C) was used in all experiments. Prior to use, 5,5‐dimethyl‐1‐pyrroline *N*‐Oxide (DMPO, Sigma–Aldrich Co. LLC., purity ≥97.0%) was purified by sublimation under reduced pressure. (T‐4)‐Difluoro[5‐[[5‐[(1E,3E)‐4‐phenyl‐1,3‐butadien‐1‐yl]‐2H‐pyrrol‐2‐ylidene‐κN]methyl]‐1H‐pyrrole‐2‐undecanoato(2‐)‐κN1]‐borate(1‐), monohydrogen (C11‐BODIPY) was purchased from Dojindo Laboratory (Kumamoto, Japan). Fatal bovine serum (FBS, E.U. origin) for cell culture was acquired from Biowest (France). Acryloxyethyl thiocarbamoyl Rhodamine B was also purchased from Polysciences Inc. (Warrington, USA). Other reagents were used as received.

### 2.2 Synthesis of Sulfobetaine Methacrylate (SBMA)

SBMA was prepared as previously reported [29, 31, 34]. Briefly, 1,3‐propanesultone 5 mL (56.9 mmol) dissolved in acetone 50 mL was added slowly dropwise to 2‐(dimethylamino)ethyl methacrylate (10 mL, 59.5 mmol) dissolved in acetone 50 mL in an iced bath under an Ar atmosphere. The reaction mixture was stirred at room temperature for 48 h in the dark. The solid product was filtered, and the filtrate was recrystallized from MeOH and acetone to yield colorless crystals (14.3 g, 90.0% yield), as shown in Figure S1.

^1^H NMR [500 MHz, D_2_O, δ = 0 ppm (tetramethylsilane (TMS))]: 6.05 (s, 1H; ─C═CH_2_); 5.66 (s, 1H; ─C═CH_2_); 4.53–4.54 (t, 2H; COO─CH_2_−); 3.70–3.72 (t, 2H; ─CH_2_─SO_3_^−^); 3.52–3.56 (t, 2H; ─^+^N(CH_3_)_2_─CH_2_─); 3.21 (s, 6H; ─^+^N(CH_3_)_2_─); 2.85–2.88 (t, 2H; ─CH_2_──^+^N(CH_3_)_2_─); 2.18–2.26 (br m, 2H; ─CH_2_─CH_2_─CH_2_─); 1.88 (s, 3H; ─CH_3_).

### 2.3 Synthesis of *N*‐(*p*‐Aminophenethyl)Methacrylamide (APEMA)

APEMA was prepared as previously reported [31]. Briefly, 2‐(4‐aminophenyl)ethylamine 1.00 g (7.34 mmol) and TEA 2.23 g (22.0 mmol) were dissolved in DCM 20 mL. The solution was cooled in an ice bath and then methacryloylchloride 0.77 g (7.34 mmol) dissolved in DCM 50 mL was added slowly dropwise to the solution under a nitrogen atmosphere. The reaction mixture was stirred at room temperature, and the reaction progress was monitored by thin layer chromatography with DCM:MeOH = 10:1 as the eluent. After stirring for 12 h, the reaction mixture was filtered. The filtrate was extracted with brine 200 mL and DCM 200 mL. The separated organic phase was dried over MgSO_4_, and the filtrate was evaporated under reduced pressure. The resulting residue was purified by silica‐gel column chromatography (DCM:MeOH = 5:1) to give APEMA (white solid, 450 mg, 30% yield), as shown in Figure S2.

^1^H NMR [500 MHz, CDCl_3_, δ = 0 ppm (TMS)]: 6.97 (d, 2H; Ar H), 6.64 (d, 2H; Ar H), 5.86 (s, 1H; ─CONH─), 5.61 (s, 1H; ─C═CH_2_), 5.26 (s, 1H, ─C═CH_2_), 3.64 (s, 2H; Ph─NH_2_─), 3.49 (t, 2H; CONH─NH_2_), 2.73 (t, 2H; Ph─CH_2_─), 1.91 (s, 3H; ═C─CH_3_).

### 2.4 Synthesis of Artesunate‐Conjugated APEMA (ART‐ethyl‐MA)

ART‐ethyl‐MA was also prepared as previously described [31]. Briefly, artesunate 1.53 g (3.97 mmol), APEMA 600 mg (2.94 mmol), and DMAP 78.2 mg (0.64 mmol) were dissolved in DCM 20 mL. The solution was cooled in an ice bath and then EDC‐HCl 830 mg (4.33 mmol) diluted with DCM 50 mL was added slowly dropwise to the solution. The reaction mixture was stirred at room temperature, and the reaction progress was monitored by thin layer chromatography. After stirring for 12 h, the reaction mixture was filtered. The filtrate was extracted with brine 200 mL and DCM 200 mL. The separated organic phase was dried over Na_2_SO_4_, and the filtrate was evaporated under reduced pressure. The resulting residue was purified by silica‐gel column chromatography (*n*‐hexane:ethyl acetate = 4:1) to give ART‐ethyl‐MA (white solid, 1,020 mg, 60.8% yield), as shown in Figure S3 and S4.

^1^H NMR [500 MHz, CDCl_3_, δ = 0 ppm (TMS)]: 8.05 (s, 1H; Ph─NHCO), 7.50 (d, 2H; Ar H), 7.11 (d, 2H; Ar H), 5.90 (s, 1H; ═C─CONH), 5.79 (d, 1H; ─O─CH─O─, H10), 5.61 (s, 1H; ─C═CH_2_), 5.44 (s, 1H; ─O─CH─O─, H11), 5.30 (s, 1H; ─C═CH_2_), 3.52 (q, 2H; CONH─CH_2_─), 2.53–2.85 (m, 6H; ─CH_2_─COO─, Ph─CONH─CH_2_─, ─CH_2_─Ph, H19, H20), 2.37 (td, 1H; ─CH─, H8a), 2.05 (m, 1H; ─CH─, H5a), 1.92 (m, 4H; ─CH_2_─CH─, ═C─CH_3_, H6), 1.60–1.80 (m, 3H, ─CH─, ─CH_2_−, H9, H4), 1.25–1.42 (m, 9H; ─CH_2_─, ─CH_2_─, ─CH_2_─, ─CH─CH_3_, H5, H7, H8, H14), 0.97 (d, 3H; ─CH_3_, H16), 0.85 (d, 3H; ─CH_3_, H15).

### 2.5 Solid‐State Copolymerization for the Synthesis of PSBMA‐ARTs Conjugates

Typically, solid‐state copolymerization was carried out with the Amalgam mixer D (Shofu Inc., Kyoto, Japan) equipped with an SUS twin‐shell blender (7.8 mmΦ, 24 mm long) and an SUS ball (6.0 mmΦ, 890 mg) under a nitrogen atmosphere at room temperature. Residual oxygen was removed using a Model 1000 Oxygen Trap (3001‐17905, GL Science Inc., Japan) and the oxygen concentration was monitored with an oxygen analyzer (LC750/PC‐120, Toray Engineering Co., Ltd., Japan) to ensure that it remained below 0.01 ppm. All sample manipulations were performed in a vacuum glove box (Sanplatec Corp., Japan) under a nitrogen atmosphere. The mixtures of SBMA (73.5 mg, 85 mol%) and ART‐ethyl‐MA (26.5 mg, 15 mol%) were encapsulated in a SUS jar and ball, and the ball‐milling at a frequency of 60 Hz was performed for 7, 10, 15, 30, and 60 min of the copolymerization under the above conditions. For comparative evaluation in the LDH assay, solid‐state polymerization of SBMA (100 mg, 100 mol%) and solid‐state copolymerization of SBMA (90.0 mg, 85 mol%) and *N*‐benzylmethacrylamide (BnMA, 10.0 mg, 15 mol%) were carried out to prepare PSBMA and PSBMA‐*b*‐PBnMA, according to previously reported [30]. The fluorescence‐labeled PSBMA‐ARTs conjugates to investigate cellular uptake were fabricated by the solid‐state copolymerization of SBMA (73.3 mg, 85 mol%), ART‐ethyl‐MA (24.7 mg, 14 mol%), and acryloxyethyl thiocarbamoyl Rhodamine B (2.0 mg, 1 mol%). Each resulting polymer was dialyzed with water and MeOH to remove each remaining monomer, and the inner layer obtained was freeze‐dried and then used in the following evaluations.

### 2.6 Characterization

#### 2.6.1 ^1^H‐NMR Measurements

^1^H‐NMR spectra were recorded on a JEOL ECA500 FT‐NMR spectrometer in D_2_O, MeOH‐*d*_4_, or TFE‐*d*_2_ containing maleic acid (1.0% (w/v)) as a quantification standard and TMS (0.1% (w/v)) as an internal standard. The resulting samples obtained from solid‐state copolymerization were exposed to air to quench the radicals before their ^1^H‐NMR spectra were measured. Polymer conversion was estimated based on the decrease in the olefinic proton peak intensity of the monomer compared with a constant signal provided by a known quantity of maleic acid. Measurements were obtained at 50°C to avoid the effect of the upper critical solution temperature in the produced PSBMA. The reduction rate of the integral value of the olefinic proton peak of unpolymerized SBMA and ART‐ethyl‐MA was calculated using the following Equation (1), and the polymer conversion was evaluated:

(1) $$\begin{array}{cc} & \mathrm{Polymer}\;\mathrm{conversion}\;\left(\%\right)\\ & =\left(1-\frac{\mathrm{Integral}\;\mathrm{value}\;\mathrm{of}\;\mathrm{each}\;\mathrm{olefinic}\;\mathrm{proton}\;\mathrm{peak}\;\mathrm{after}\;\mathrm{copolymerization}}{\mathrm{Integral}\;\mathrm{value}\;\mathrm{of}\;\mathrm{each}\;\mathrm{olefinic}\;\mathrm{proton}\;\mathrm{peak}\;\mathrm{before}\;\mathrm{copolymerization}}\right)\\ & \;\times 100 \end{array}$$

#### 2.6.2 Size Exclusion Chromatography (SEC) Measurements

The molecular weights of the resulting polymers were determined by SEC at 40°C (flow rate: 0.7 mL min^−1^) in pH 7.4 phosphate‐buffered saline (PBS) containing 100 mM NaCl as an eluent. Three kinds of polyvinylalcohol gel columns (Asahipak GF‐1G 7B: particle size = 9 µm; 7.5 i.d. × 50 mm; Asahipak GS‐320 HQ: exclusion limit = 4.0 × 10^4^, particle size = 5 µm; 7.5 i.d. × 300 mm; and Asahipak 7M‐HQ: exclusion limit = 1.0 × 10^8^; particle size = 9 µm; 7.5 i.d. × 300 mm) were connected to a PU 880 high‐performance liquid chromatography (HPLC) unit (GL Sciences Inc., Japan) equipped with a RI‐2000 refractive index detector (Lab system Co., Ltd, Japan), a model 554 LC column oven (GL Sciences Inc., Japan), and a data analyzer (Runtime Instruments Chromato‐PRO, Runtime Instruments Ltd, Japan). The system was calibrated with pullulan standards (Shodex; peak molecular weight (M_p_) = 5,900, 9,600, 21,100, 47,100, 107,000, 194,000, 337,000, and 642,000 g mol^−1^).

#### 2.6.3 HPLC Measurements

The stability test of the resulting polymers were evaluated by HPLC using the following equipment and parameters: a Hewlett‐Packard HPLC system (Hewlett‐Packard, Agilent 1100, Palo Alto, USA) equipped with an Agilent 1100 Series autosampler and Hewlett‐Packard Agilent 1100 data analysis software (Agilent ChemBion); an excitation wavelength of 200 nm; a binary phase in an InertSustain C18 analytical column (particle diameter of 5 µm, 4.6 × 250 mm) (GL sciences); a mobile phase comprising a EtOH/10 mM formic acid aqueous solution mixture (65:35, v/v%); a flow rate of 0.5 mL min^−1^; an injection volume of 20 µL; and a column temperature of 50°C.

#### 2.6.4 Dynamic Light Scattering (DLS) Measurements

DLS measurements (DLS‐8000DSL, Otsuka Electronics, Japan) were performed using a He/Ne laser (λ_0_ = 633 nm) at 25°C in pH 7.4 PBS to calculate the diffusion coefficient *D* using the cumulant method and Equations (2) and (3).

(2) $$\Gamma =q^{2}\;D$$

(3) $$q=\frac{4\pi n_{0}}{\lambda_{0}}\;sin\left(\frac{\theta }{2}\right)$$

(4) $$q^{2}=\frac{16\pi^{2}{n_{0}}^{2}}{{\lambda_{0}}^{2}}\;sin^{2}\left(\frac{\theta }{2}\right)$$

where Г, *q*, n_0_, λ_0_, and *θ* are the attenuation constant, scattering vector, refractive index of the solvent, wavelength of the laser light, and scattering angle, respectively. The hydrodynamic diameter *d_h_* was calculated from the diffusion coefficient *D* at a scattering angle of 90° using the Einstein–Stokes equation (Equation 5).

(5) $$d_{h}=\frac{k_{B}T}{3\pi \eta_{0}D}\;$$

where *k_B_*, T, and *η_0_* are the Boltzmann's constant, absolute temperature, and solvent viscosity, respectively. The number distribution of particle size of the samples was also determined using the histogram method with Contin's calculation. All sample solutions used in the measurements were filtered through a 0.8‐µm membrane filter (DISMIC‐25CS, ADVANTEC, Japan).

### 2.7 Free Radical Productivity of PSBMA‐ARTs Conjugates Using ESR

Free radical generation from resulting PSBMA‐ARTs conjugates was confirmed by ESR measurements using sublimated DMPO as a spin‐trapping agent. The ESR measurement was performed at room temperature according to previous reports [35, 36, 37, 38, 39, 40, 41, 42]. Briefly, 45.3 mg of DMPO was dissolved in 50% (v/v) D_2_O in pure water to prepare 1 mL of 400 mM DMPO aqueous solution. Fe(NH_4_)_2_(SO_4_)_2_·6H_2_O (0.8 mg) was also dissolved in 50% (v/v) D_2_O in pure water to prepare 1 mL of 4 mM ferrous ion aqueous solution. Artesunate (1.54 mg) was dissolved in 50 µL of DMSO, and diluted up to 1 mL using 50 v/v% D_2_O in pure water to prepare a 4 mM aqueous solution of artesunate. PSBMA‐ARTs conjugates aqueous solution at 4 mM was similarly prepared with 8.62 mg of PSBMA‐ARTs conjugates containing 1.54 mg of artesunate and 50 v/v% D_2_O in pure water. DMPO could be oxidized by free radicals generated, and the spin‐trapped radicals were observed in ESR measurements. To investigate the spin‐trapping of the generated radicals from artesunate or PSBMA‐ARTs conjugates, 4 mM artesunate or 4 mM PSBMA‐ARTs conjugates as artesunate moieties, 4 mM ferrous ion, and 400 mM DMPO aqueous solution were quickly mixed in equal amounts, and 500 µL of the mixture was immediately taken into an ESR quartz flat cell (ES‐LC12; JEOL, Ltd., Tokyo, Japan). The ESR flat cell was placed in the cavity of the spectrometer, and the ESR spectra were recorded over time. ESR spectra were recorded on a JES‐FA200 (JEOL Ltd., Japan) spectrometer with X‐band and 100 kHz field modulation. Care was taken to ensure that no saturation occurred and that the line shape was not distorted by excessive modulation amplitude. From a plot of the square root of the microwave power versus the signal peak height, a microwave power level of 4 mW was chosen. The ESR spectral intensity was determined by double integration. The magnetic field was set at 336.8 mT; field sweep, 7 mT; modulation amplitude, 1.0 G; time constant, 300 ms; sweep time, 8 min. The hyperfine splitting constants (Hfc) were calculated by the splitting of MnO (_Δ_H = 8.69 mT).

### 2.8 Protein Adsorption Property of PSBMA‐ARTs Conjugates

Albumin at 10% (w/v), fibrinogen at 0.6% (w/v), and 5 mg mL^−1^ of aqueous solutions of PSBMA‐ARTs conjugates were prepared with pH 7.4 PBS and filtered through a 0.8‐µm filter. The aqueous solution of PSBMA‐ARTs conjugates and that of albumin or fibrinogen were mixed and incubated at 37°C for 4 h. DLS measurements were performed on the samples before and after incubation to evaluate the changes in particle size of PSBMA‐ARTs conjugates.

### 2.9 Stability Test of PSBMA‐ARTs Conjugates Using Hydrolases

To evaluate the stability of PSBMA‐ARTs conjugates under physiological conditions, hydrolysis tests were conducted using lipase from porcine pancreas (Lot No. AVZ6A‐JI, Tokyo Chemical Industry Co., Ltd, Tokyo, Japan) at concentrations equivalent to those in blood. Briefly, 270 mg PSBMA‐ARTs conjugates (45.9 mg as artesunate) and 4.5 U lipase were dissolved in 5 mL of pH 7.4 PBS, and the solution was transferred to a dialysis membrane tube (Spectra/Por7, MWCO 1,000) and immersed in 40 mL of pH 7.4 PBS as the outer layer. The solution was incubated at 37°C, and 500 µL of the outer layer was collected at predetermined time points, and then 500 µL of fresh pH 7.4 PBS was added to the outer layer. The collected samples were analyzed with HPLC, and the artesunate released via hydrolysis of the side chains of the PSBMA‐ARTs conjugates was evaluated using a calibration curve method.

### 2.10 Cell Culture

The human hepatoblastoma cell lines (HepG2) was purchased from Cellular Engineering Technologies, Inc. (Crosspark Rd, Coralville IA, USA). HepG2 cells were grown in Eagle's minimum essential medium (E‐MEM) supplemented with 10% heat‐inactivated fetal bovine serum (FBS), 100 U mL^−1^ penicillin, 100 µg mL^−1^ streptomycin, and 2 mM L‐glutamine at 37°C under 5% CO_2_ in a humidified incubator. The cells were passaged by trypsinization with 0.25% trypsin/EDTA every 3～5 days, and were used in all subsequent experiments.

The primary human hepatocytes (PHH, Batch number HEP187738) were obtained from ReproCELL Inc. (Kanagawa, Japan). The PHH cell suspensions were seeded into collagen coated 96‐well plates (5 × 10^5^ cells/well) or 35 mm Petri dish (1 × 10^5^ cells/mL) with William's E medium (Gibco, Karlsruhe, Germany) containing 11.1 mM glucose, 5% (v/v) FBS, 1 µM dexamethasone DMSO solution (10 mM), 100 U mL^−1^ penicillin, 100 µg mL^−1^ streptomycin, 4 µg mL^−1^ human recombinant insulin, 2 mM L‐glutamine, and 15 mM HEPES (pH 7.4). After 16 h of incubation at 37°C under 5% CO_2_ in a humidified incubator, the medium was replaced to William's E medium for maintenance containing 11.1 mM glucose, 0.125% (v/v) FBS, 0.1 µM dexamethasone DMSO solution (10 mM), 100 U mL^−1^ penicillin, 100 µg mL^−1^ streptomycin, 6.25 µg mL^−1^ human recombinant insulin, 6.25 µg mL^−1^ human transferrin, 6.25 µg mL^−1^ selenous acid, 5.35 µg mL^−1^ linoleic acid, 2 mM L‐glutamine, and 15 mM HEPES (pH 7.4), and incubated at 37°C under 5% CO_2_ in a humidified incubator. The medium was daily replaced to fresh one until the cells were used for each evaluation.

### 2.11 Cell Viability Assay

HepG2 cell suspensions (5.0 × 10^4^ cells/well) were seeded in 96‐well plates and then incubated for 48 h at 37°C under 5% CO_2_ in a humidified incubator. Fe(NH_4_)_2_(SO_4_)_2_·6H_2_O (1.6 mg) and FeCl_3_·6H_2_O (1.1 mg) were separately dissolved in 2 mL of distilled water to prepare a 2 mM Fe^2+^ and Fe^3+^ aqueous solution. The medium was replaced with 90 µL of fresh medium, or 80 µL of fresh medium and 10 µL of 2 mM Fe^2+^ or Fe^3+^ aqueous solution. Artesunate 10 µL or PSBMA‐ARTs conjugates aqueous solution 10 µL prepared at each concentration was administered to each well, and the 96‐well plate was incubated for 48 h at 37°C under 5% CO_2_ in a humidifier. For the evaluation of cytotoxicity of HepG2 pretreated with ferroptosis‐ and divalent iron inhibitors, the cells were cultured in 96‐well plates until confluent. The medium was replaced with 90 µL of fresh medium, and 10 µL of either deferoxamine aqueous solution 200 µM as a final concentration or ferrostatin‐1 aqueous solution 1 µM as a final concentration was added. The mixture was then incubated for 4 h at 37°C under 5% CO_2_ in a humidifier. The medium was then removed, and the cells were quickly washed three times with pH 7.4 PBS. Subsequently, 90 µL of fresh medium was added, and 10 µL of artesunate or PSBMA‐ARTs conjugates aqueous solution were administered in the same manner to the above protocol.

2‐(4‐Iodophenyl)‐3‐(4‐nitrophenyl)‐5‐(2,4‐disulfophenyl)‐2*H*‐tetrazolium sodium salt (WST‐1, Dojindo Laboratory, Kumamoto, Japan) was dissolved in 20 mM pH 7.4 HEPES buffer to prepare a 5.5 mM WST‐1 solution, and 1‐methoxy‐5‐methylphenazinium methylsulfate (1‐methoxy PMS, Dojindo Laboratory, Kumamoto, Japan) was dissolved in sterile water to prepare a 2 mM 1‐methoxy PMS solution. Then, 5.5 mM WST‐1 and 2 mM 1‐methoxy PMS solution were mixed at a ratio of 19: 1 and then filtered through a 0.22‐µm sterile membrane filter (Millex‐GV Syringe Filter Unit, Millipore, USA). WST‐1 reagent solution (10 µL) was added to each well, and the plate was incubated for 2 h at 37°C under 5% CO_2_ in a humidified incubator. The absorbance of each well was measured at 450 nm. The percentage of cell viability was calculated using Equation (6).

(6) $$\mathrm{Cell}\;\;\mathrm{viability}\;\left(\%\right)=\left[\frac{\left(\mathrm{Ab}s_{\cdot \mathrm{Experimental}}-\mathrm{Ab}s_{\cdot \mathrm{Negative}\;\;\mathrm{control}}\right)}{\left(\mathrm{Ab}s_{\cdot \mathrm{Positive}\;\mathrm{control}}-\mathrm{Ab}s_{\cdot \mathrm{Negative}\;\mathrm{control}}\right)}\right]\times 100$$

where: Abs._Experimental_ is the absorbance of each sample, Abs._Negative control_ is the absorbance without cells, and Abs._Positive control_ is the absorbance of the confluent cells. The cytotoxicity of PSBMA‐ARTs conjugates against HPP cells was evaluated as follows: 96‐well plates (cells seeded at 5 × 10^5^ cells/well) were incubated for 2 days, and treated with each polymer solution dissolved in culture medium, and the cytotoxicity was evaluated using the same WST‐1 procedure as described above.

### 2.12 Lactate Dehydrogenase (LDH) Assay

HepG2 cell suspensions (5 × 10^4^ cells/well) were seeded in 96‐well plates and then incubated for 24 h at 37°C under 5% CO_2_ in a humidified incubator. Artesunate (1.54 mg), PSBMA‐ARTs conjugates (8.62 mg), PSBMA (10 mg), and PSBMA‐*b*‐PBnMA (10 mg) were separately diluted to 1 mL with pH 7.4 PBS to prepare the sample solution at 4 mM as artesunate. The medium was replaced with 90 µL of fresh medium, and 10 µL of aqueous solution of artesunate at 400 µM as the final concentration, PSBMA‐ARTs conjugates at 400 µM as artesunate moiety, PSBMA at 1 mg mL^−1^, and PSBMA‐*b*‐PBnMA at 1 mg mL^−1^ were separately added, and then incubated for 24 h at 37°C under 5% CO_2_ in a humidified incubator. LDH assay buffer was prepared according to the manual of the Cytotoxicity LDH Assay Kit‐WST (Dojindo Laboratory, Kumamoto, Japan) to measure LDH released from cells. The supernatant (50 µL) was transferred to new 96‐well plates, and 10 µL of lysis buffer was added to each well and incubated for 30 min at 37°C under 5% CO_2_ humidifier. The working solution (100 µL) was added to each well, and then incubated for 30 min at room temperature, followed by the addition of stop solution (50 µL). The absorbance of each well was measured at 490 nm using a microplate reader (GloMax Discover, Promega, Madison, WI, USA). The LDH release rate was calculated with the absorbance of the lysis buffer as 100%.

### 2.13 Confocal Laser Scan Microscopy (CLSM)

The lipid‐peroxidation induced by artesunate or PSBMA‐ARTs conjugates in HepG2 cells was visualized by CLSM with C11‐BODIPY, and the brightness analysis of green fluorescence of C11‐BODIPY was evaluated to determine the extent of lipid‐peroxidation. After preparing 2.0 mL of medium in a 35 mm petri dish, 1.0 mL of HepG2 cell suspensions (2.0 × 10^4^ cells mL^−1^) were seeded into the dish, and the cells were incubated for 24 h at 37°C under 5% CO_2_ in a humidified incubator. Artesunate dissolved in 0.1% (v/v) DMSO aqueous solution at 40 µM and PSBMA‐ARTs conjugates dissolved in pH 7.4 PBS at 40 µM as artesunate moiety were prepared, and filtered through a sterilized membrane filter with 0.80‐µm pores (Dismic25CS, Advantec, Tokyo, Japan). The medium was replaced with 1.8 mL of fresh medium, and 200 µL of the above aqueous solution was added, respectively. The cells were incubated for 24 h at 37°C in a 5% CO_2_ humidifier. C11‐BODIPY aqueous solution was prepared according to the manual of Lipid Peroxidation Probe‐BDP 581/591 C11 (Dojindo Laboratory, Kumamoto, Japan). C11‐BODIPY for 200 measurements in a vial was dissolved with 20 µL DMSO to prepare a stock solution. This stock solution was diluted 2,000‐fold using serum‐free E‐MEM and used as the working solution. After removal of the medium, the cells were quickly washed three times with pH 7.4 PBS, and the nucleus was stained with an aqueous solution of Hoechst33342 (Invitrogen/Thermo Fisher Scientific, Grand Island, NY USA) for 10 min of incubation. After removal of the aqueous solution for nucleus staining, the cells were quickly washed three times with pH 7.4 PBS. The working solution prepared (2 mL) was added to the cells and incubated for 30 min, and the cells were quickly washed three times with pH 7.4 PBS. After removal of the aqueous solution, 2.0 mL of FluoroBrite^TM^ in Dulbecco's Modified Eagle Medium (D‐MEM, Thermo Fisher Scientific, Waltham, MA, USA) was added to the cells. The Fluorescence images were acquired with a Zeiss LSM 900 confocal microscope. Zen software (Zeiss, Germany) was used for image processing.

The cellular uptake of Rhodamine B‐labeled PSBMA‐ARTs conjugates was investigated in the same manner using CLSM. Each cell was treated with the following procedure. After preparing 2.0 mL of medium in a 35 mm Petri dish, 1.0 mL of cell suspensions (2.0 × 10^4^ cells mL^−1^) were seeded into the dish, and the cells were incubated for 24 h at 37°C under 5% CO_2_ in a humidified incubator. Each aqueous solution of Rhodamine B‐labeled PSBMA‐ARTs conjugates was prepared at 250 µg mL^−1^ as Rhodamine B with pH 7.4 PBS, and filtered through a sterilized membrane filter with 0.80‐µm pores. The medium was replaced with 1.8 mL of fresh medium, and 200 µL of the above polymer aqueous solution was added (the final concentration of the polymer was 25 µg mL^−1^ as Rhodamine B), and the cells were incubated for a predetermined time at 37°C in a 5% CO_2_ humidifier. After removal of the medium, the cells were washed three times with pH 7.4 PBS, and stained with an aqueous solution of Hoechst33342 for 10 min of incubation. After removal of the aqueous solution for nucleus staining, the cells were washed three times with pH 7.4 PBS, and 2.0 mL of FluoroBrite D‐MEM was added to the cells.

For energy dependence and inhibition of endocytosis tests, HepG2 cells seeded at the same concentration as above in a 35 mm Petri dish were pre‐incubated for 30 min with each endocytosis inhibitor at 37°C, or pre‐incubated for 60 min at 4°C, prior to administration of Rhodamine B‐labeled PSBMA‐ARTs conjugates. Each endocytosis inhibitor was utilized at the following concentration: amiloride 11.5 µg mL^−1^, chlorpromazine hydrochloride 10 µg mL^−1^, dynasore 25.8 µg mL^−1^, methyl‐β‐cyclodextrin 2.0 mg mL^−1^, and sodium azide 650 µg mL^−1^. Each result of cellular uptake was expressed with brightness analysis of Rhodamine B‐labeled PSBMA‐ARTs conjugates internalized into cells, compared to the control.

## 3 Results and Discussion

### 3.1 Synthesis and Characterization of PSBMA‐ARTs Conjugates

**FIGURE 2:**
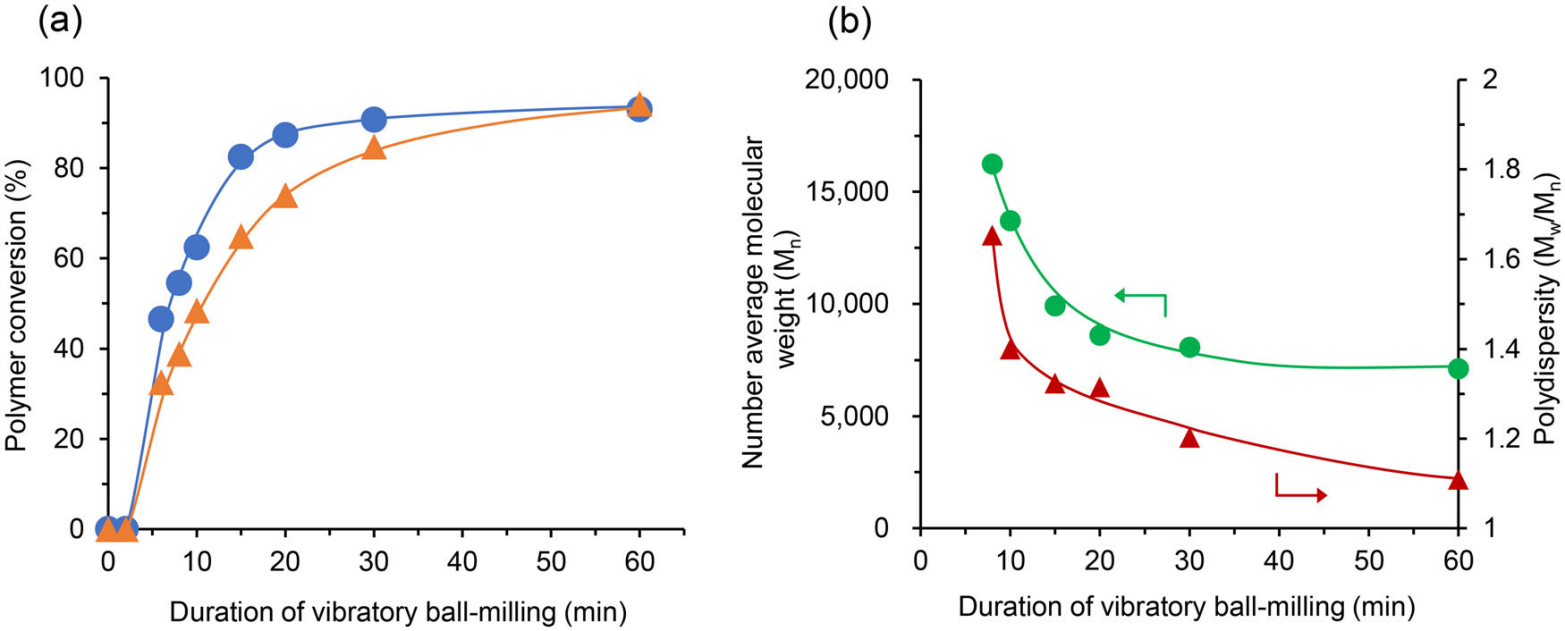
Progressive changes in the polymer conversion rate of SBMA (circle) and ART‐ethyl‐MA (triangle) during solid‐state copolymerization (a), and molecular weight (circle) and polydispersity (triangle) of resulting polymers synthesized by the copolymerization (b).

The liquid‐phase copolymerization of zwitterionic monomer and hydrophobic monomer has enthusiastically been attempted in the past decade [43, 44, 45, 46, 47, 48, 49]. However, their copolymerization might not progress in the liquid phase, due to significant differences in the monomer solubility and polymer precipitation during copolymerization. Although 2‐methacryloyloxyethyl phosphorylcholine mimicking the molecular structure of phosphatidylcholine, which constitutes cellular membranes, could successfully be copolymerized with *n*‐butyl methacrylate in alcohol solution [50, 51, 52, 53], the copolymerization of zwitterionic and a hydrophobic monomer possessing a bulky side chain has not yet been reported. Therefore, solid‐state polymerization methods, which do not require consideration of the affinity between monomers and solvents or catalysts, would be an innovative advantage for the copolymerization of zwitterionic monomers and hydrophobic monomers. Then, we carried out the solid‐state copolymerization of SBMA and ART‐ethyl‐MA, and the characterization of the resulting polymer was investigated. The solid‐state copolymerization of 85 mol% SBMA and 15 mol% ART‐ethyl‐MA was conducted at room temperature under a nitrogen atmosphere for each copolymerization time. The polymer conversion of SBMA and ART‐ethyl‐MA was determined by ^1^H‐NMR, as shown in Figure 2a.

The olefinic proton peak intensity of each monomer decreased with time, and the polymer conversion rate was achieved more than 90% after 60 min of copolymerization. It also can be seen in Figure 2b that the number average molecular weight (M_n_) of resulting polymers against copolymerization time showed an exponential decrease, due to the main‐chain scission of resulting polymers caused by the mechanical energy. The polydispersity (M_w_/M_n_, PDI) of the resulting polymers also approached monodispersity with prolonged milling, since it was adjusted to a uniform molecular weight. The M_n_ and PDI of the resulting polymer synthesized by 60 min of copolymerization were 8,000 g mol^−1^ and 1.10, respectively. It can be seen in Figure S5 that the resulting polymer synthesized by 60 min of copolymerization was dialyzed with MeOH to remove unpolymerized monomers as the external phase, followed by dialysis with distilled water and freeze‐drying of the internal phase to yield (PSBMA)_21_‐*b*‐(PolyART‐ethyl‐MA)_4_. The composition of the resulting polymer was consistent with the monomer preparation ratio before copolymerization. Furthermore, solid‐state copolymerization of 85 mol% SBMA and 15 mol% ART‐ethyl‐MA also conducted at a reduced vibration frequency of 25 Hz, and the resulting polymer with a molecular weight of 15,900 g mol^−^^1^ and a composition identical to the monomer preparation ratio was yielded, as shown in Figure S6. Since the main‐chain scission of the resulting polymer could be controlled by varying the mechanical energy, it would be possible to adjust the molecular weight of resulting polymer.

**FIGURE 3:**
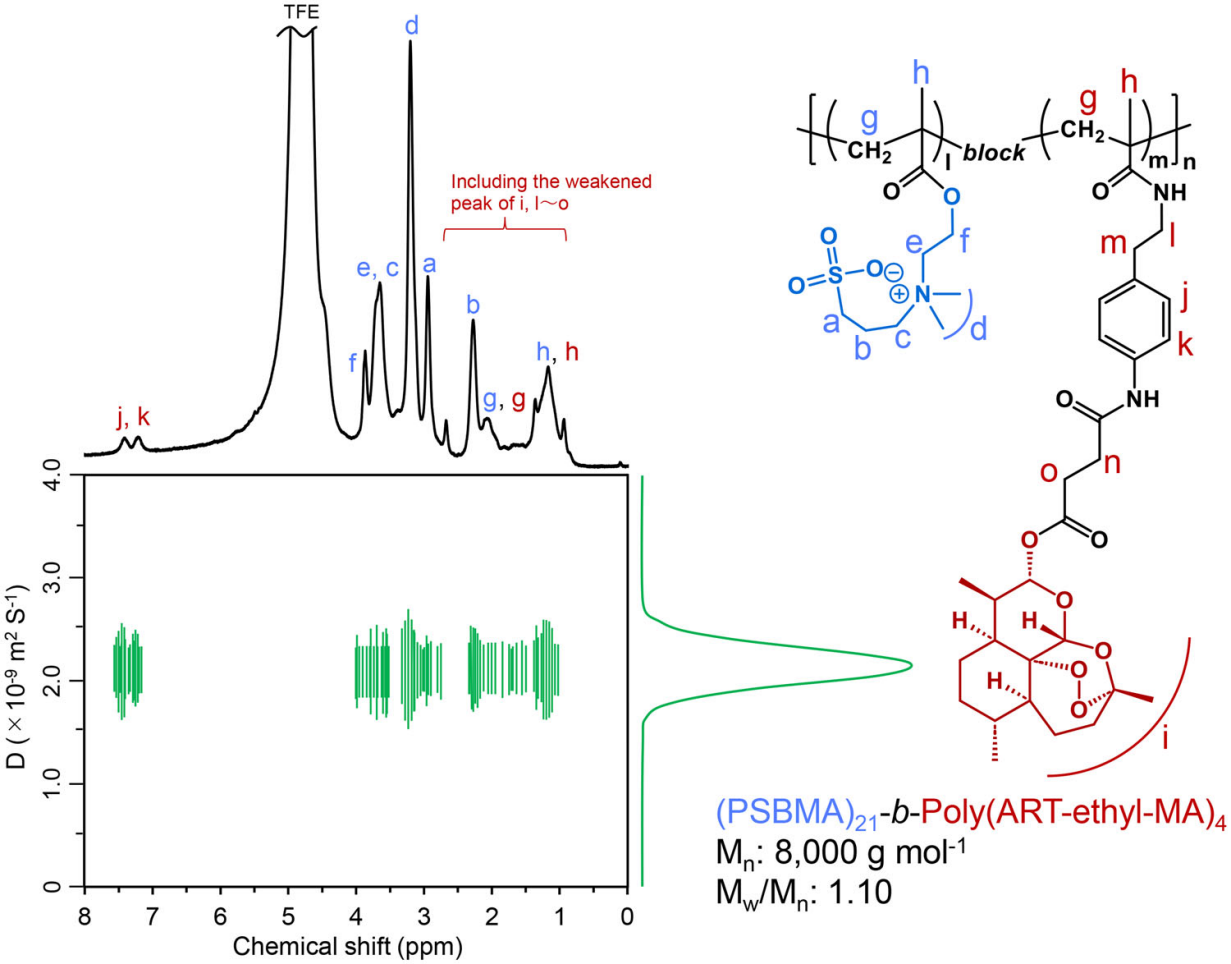
^1^H‐NMR/DOSY spectrum of purified PSBMA‐ARTs conjugates.

It can be seen in Figure 3 that the diffusion distribution of (PSBMA)_21_‐*b*‐(PolyART‐ethyl‐MA)_4_ showed a single distribution in the DOSY‐NMR. Therefore, it would be assumed that the resulting polymer synthesized by solid‐state copolymerization of SBMA and ART‐ethyl‐MA possessed a block copolymer structure in which PSBMA and poly(ART‐ethyl‐MA) were linked. The (PSBMA)_21_‐*b*‐(PolyART‐ethyl‐MA)_4_ was utilized as PSBMA‐ARTs conjugates in the following experiment.

### 3.2 ESR Measurement for Dose‐Dependent Free Radical Productivity of PSBMA‐ARTs Conjugates

**FIGURE 4:**
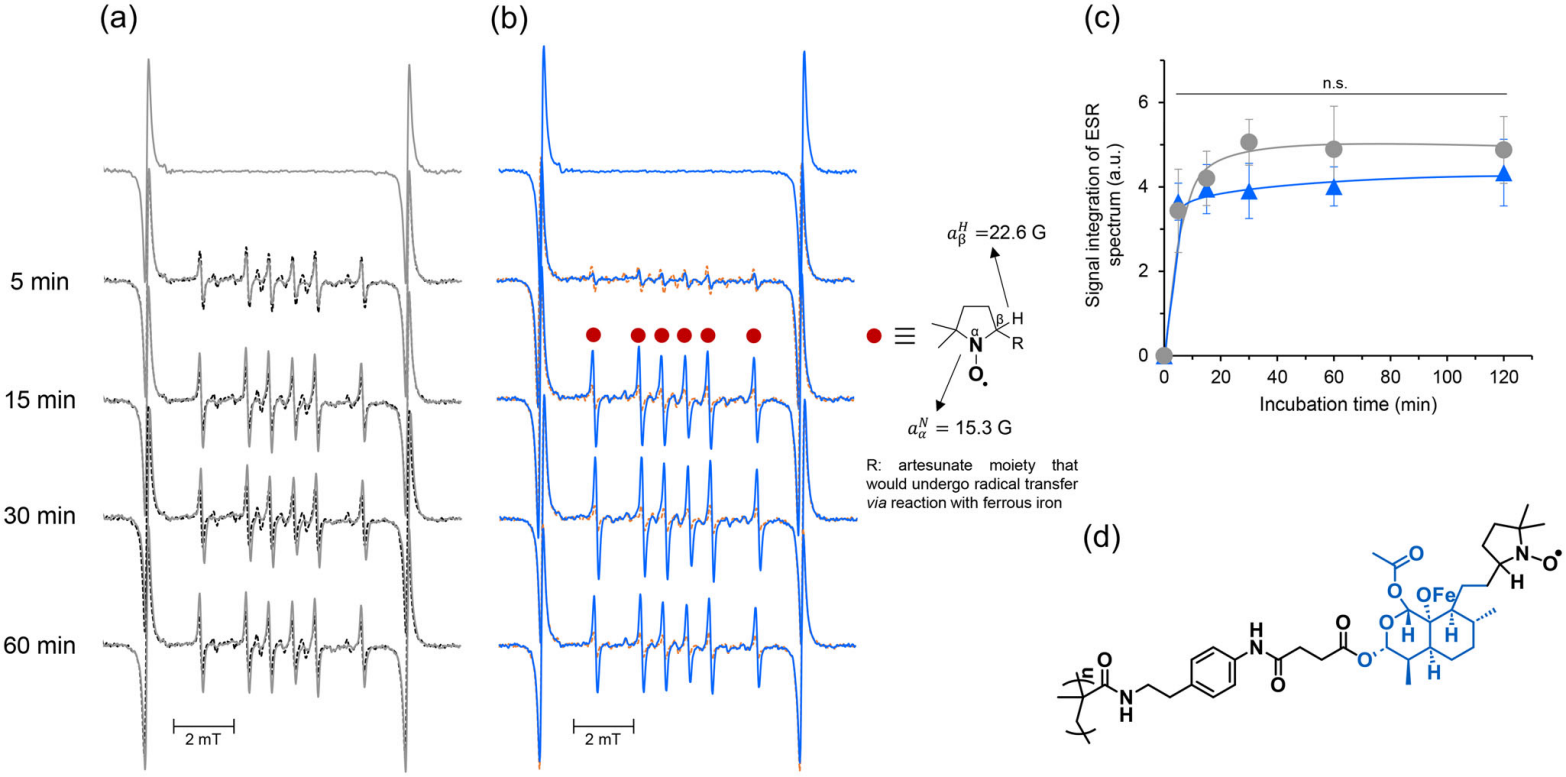
ESR spectra and their spectrum simulations as dotted lines of DMPO spin adducts generated from artesunate (a) or PSBMA‐ARTs conjugates (b) and the incubations with ferrous ion. Progressive changes in signal integration of ESR spectrum (c). The circle and triangle indicate artesunate and PSBMA‐ARTs conjugates, respectively. Assumed side‐chain structure of artesunate‐DMPO adducts in poly(ART‐ethyl‐MA) (d). Statistical significance in (c) was measured between two groups using unequal variances Welch's t‐test.

5,5‐Dimethyl‐1‐pyrroline *N*‐Oxide (DMPO) is a profitable tool for trapping free radicals, and the characteristic spectrum would be observed by ESR measurement. The free radical productivity of PSBMA‐ARTs conjugates was examined with DMPO. Although each 4 mM PSBMA‐ARTs conjugates as artesunate moiety, 4 mM artesunate, and 4 mM ferrous ion aqueous solution was separately added to 400 mM sublimated DMPO aqueous solution, no ESR spectrum was detected either. It can be seen in Figure 4a,b that the triplet of doublet spectra were confirmed in the reaction mixture of artesunate or PSBMA‐ARTs conjugates and ferrous ion.

The DMPO adducts generated by spin trapping of a free radical can undergo a three‐fold splitting due to coupling with nitrogen, which has a nuclear spin of 1. This triplet line spectrum should also cause a two‐fold splitting due to coupling with the β‐position hydrogen, thus exhibiting a characteristic sextet line (triplet of doublets). It is well‐known that the oxygen‐centered radical generated by the reaction between artemisinin and ferrous iron would be thermodynamically unstable and therefore cannot be spin‐trapped under any conditions [37, 38]. A. Alberti and G. Marconi et al. revealed that the radical species generated by the reaction of artemisinin and ferrous ion were carbon‐centered radicals (a_N_ = 15 G, a_H_ = 22.3 mT, and *g* = 2.0056) attributed to compound **6** through the ESR spectrum simulation of the resulting DMPO adduct, as shown in Figure S7 [36]. Moreover, HPLC‐ESR system has demonstrated that compound **3** with a oxygen‐centered radical and compound **6** with a primary carbon radical were generated in nearly equal quantities in the reaction between artesunate and ferrous iron under physiological conditions, as reported by M. Asano and H. Iwahashi [36]. Therefore, it was suggested that the DMPO adduct shown in Figure 4d would predominantly be formed thermodynamically stable radical **6** in neutral aqueous solutions at room temperature during the spin‐trapping method using DMPO, while it is entirely possible that oxygen‐centered radical **3**, which cannot be detected by ESR measurements, might be produced. The ESR spectral simulation confirmed that the DMPO adduct (a_N_ = 15.3 G, a_H_ = 22.6 G, *g* = 2.0055) of the radical generated from PSBMA‐ART conjugates corresponded well with that reported by A. Alberti and G. Marconi et al., as shown in Figure 4b [36]. It can be seen in Figure 4c that the double‐integrated values of the ESR spectra for the DMPO adducts from artesunate and those from PSBMA‐ARTs conjugates showed no significant difference regardless of the incubation time with ferrous iron. Therefore, it was revealed that the artesunate moiety introduced into the hydrophobic chain in PSBMA‐ARTs conjugates would efficiently dissolve in water and react with ferrous ions due to the hydration effect of PSBMA.

### 3.3 Protein Antifouling Effects and Stability Against Lipase of PSBMA‐ARTs Conjugates

DDS carriers administered intravenously should prevent protein adsorption, which induces embolism of capillary vessels. The particle size of DDS carrier with biocompatibility would be maintained in the presence of serum proteins, due to the antifouling effects. The protein adsorption property of PSBMA‐ARTs conjugates was investigated with DLS measurements to evaluate the changes in particle size. If the interaction between DDS carrier and serum proteins were to occur, the particle size of DDS carrier should increase. Albumin and fibrinogen were selected as serum proteins, and each aqueous solution at concentrations matching their blood levels was prepared. PSBMA‐ARTs conjugates were incubated with the aqueous solution of albumin or fibrinogen for the saturation time, and DLS measurement of each mixture sample was performed.

**FIGURE 5:**
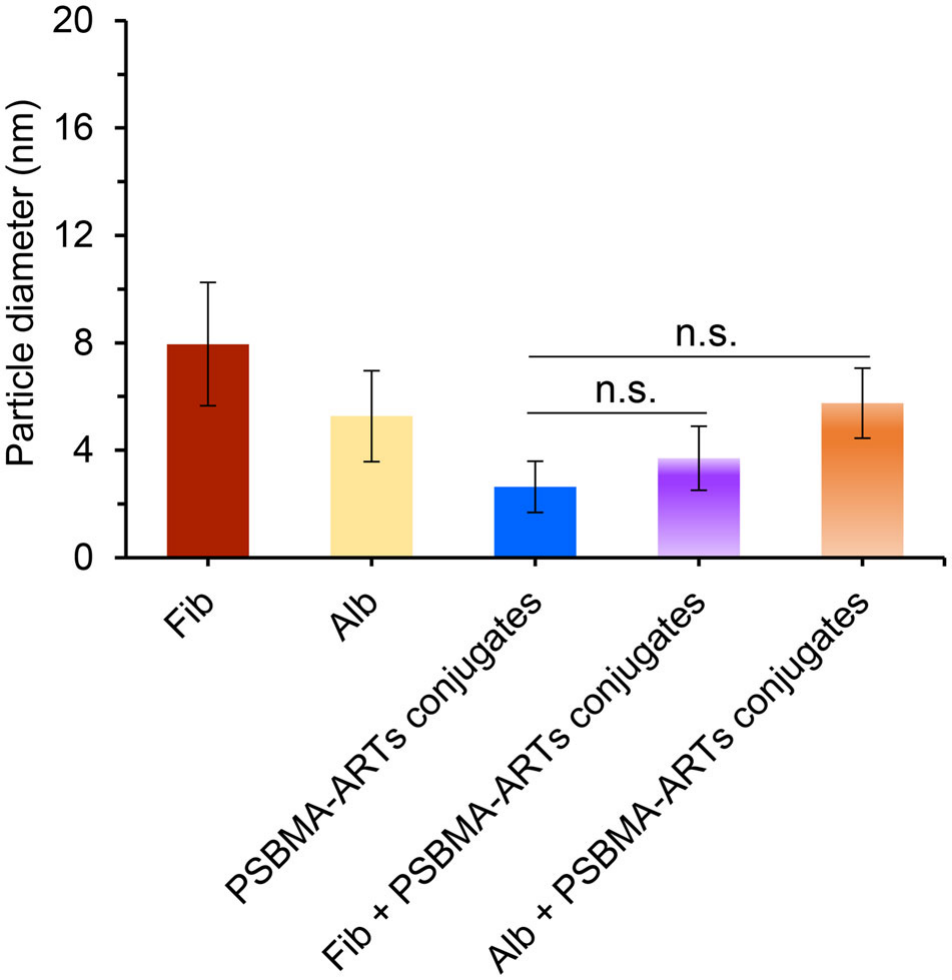
Protein antifouling property of PSBMA‐ARTs conjugates. Albumin and fibrinogen are indicated as Alb and Fib. Both data are shown as the mean ± SD (*n* = 10). Statistical significance was measured between two groups using unequal variances Welch's t‐test.

As shown in Figure 5, the particle sizes of albumin and fibrinogen as the blank were 5 and 8 nm, respectively. The particle size of PSBMA‐ARTs conjugates was 2 nm, while no significant increase in particle size was observed in the mixture with serum proteins. PSBMA‐ARTs conjugates strongly suppressed the adsorption of albumin and fibrinogen, and the particle size remained below 5 nm even after a week of incubation. As reported in previous studies, the amphiphilic PSBMA with 15 mol% poly(*N*‐benzyl methacrylamide) (PBnMA) introduced as the hydrophobic chains also maintained the particle size below 5 nm in similar experiments with albumin and fibrinogen [30]. Therefore, PSBMA introduced at least 15 mol% of hydrophobic chains, wherein the artemisinin domain was conjugated via a linker containing an aromatic moiety, may be a biocompatible polymer with sufficient exclusion volume effects.

The high stability against blood enzymes that stand in the way of reaching the tumor site is essential for DDS carrier. Since artesunate was conjugated to the side chains of PSBMA‐ARTs conjugates via an ester linkage, the ester moiety may be hydrolyzed by enzymes such as lipase in the blood. Then, the stability of PSBMA‐ARTs conjugates against lipase (100 U L^−1^) under physiological conditions was investigated using HPLC. As shown in Figure S8, no artesunate was detected from the PSBMA‐ARTs conjugates at any incubation time in the presence of lipase at the same concentration as in blood, whereas in the blank, a distinct peak attributable to artesunate was observed at 12.5 min of the elution time. Therefore, the side chains composed of PSBMA‐ARTs conjugates exhibited high stability against lipase over the course of one week. These results suggest that PSBMA‐ARTs conjugates would suppress the interaction with enzymes and proteins.

### 3.4 Anticancer Activity of PSBMA‐ARTs Conjugates

The cytotoxicity of PSBMA‐ARTs conjugates against HepG2 cells was examined with WST‐1 assay, compared to artesunate. In order to demonstrate the anticancer effect of PSBMA‐ARTs conjugates in physiological conditions, PSBMA‐ARTs conjugates and artesunate were separately administered into HepG2 in the medium containing 10% FBS. When a 0.2 mM Fe^2+^ or Fe^3+^ aqueous solution was individually added to HepG2, no cell death was observed for 3 days of incubation. The Fe^2+^ or Fe^3+^ and artesunate or PSBMA‐ARTs conjugates were simultaneously administered to the cells, or each drug was individually administered to cells pretreated with Fe^2+^.

**FIGURE 6:**
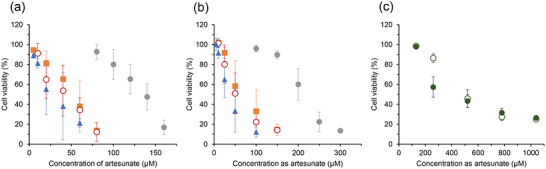
Cell viability of HepG2 treated with artesunate (a), PSBMA‐ARTs conjugates (b), and cell viability of primary human hepatocytes treated with artesunate or PSBMA‐ARTs conjugates (c). The grey circles show the cell viability treated with artesunate or PSBMA‐ARTs conjugates individually. The red‐bordered circles indicate the cell viability when each drug was individually administered to cells pretreated with Fe^2^^+^. Triangles and squares show the cell viability administered artesunate or PSBMA‐ARTs conjugates with Fe^2^^+^ or Fe^3^^+^ simultaneously. The closed and open circles in (c) represent artesunate and PSBMA‐ARTs conjugates, respectively. Both data are shown as the mean ± SD (*n* = 10). Statistical significance was measured between two groups using unequal variances Welch's t‐test.

As shown in Figure 6a,b, the IC_50_ of artesunate and PSBMA‐ARTs conjugates against HepG2 cells was 136 and 211 µM, respectively. The co‐administration of artesunate or PSBMA‐ARTs conjugates with Fe^2^^+^ resulted in the lowest IC_50_ values of 24.4 and 35.6 µM, respectively. Even when Fe^3+^ was simultaneously administered with artesunate or PSBMA‐ARTs conjugates, the IC_50_ values were significantly reduced compared to their administration alone, and indicated 51.1 and 66.4 µM, respectively. It was suggested that Fe^2+^ reduced from Fe^3+^ within the cell would react with each drug internalized into cells and produce the free radicals, thereby inducing ferroptosis. The pretreatment with Fe^2+^ made the IC_50_ values of artesunate and PSBMA‐ARTs conjugates decrease to 43.9 and 50 µM, respectively. Since each drug successfully induced cytotoxicity even in cells pretreated with Fe^2+^, PSBMA‐ARTs conjugates would effectively induce ferroptosis of cancer cells accumulating ferrous iron. In contrast, normal cells such as primary human hepatocytes (PHH) would express fewer transferrin receptors than HepG2 cells [3, 4, 5, 6, 7, 8, 54, 55, 56, 57, 58] and have lower intracellular concentrations of Fe^2+^. Therefore, PSBMA‐ARTs conjugates, which generate free radicals via reaction with Fe^2+^, may exhibit reduced cytotoxicity toward PHH cells compared to HepG2 cells. As shown in Figure 6c, the IC_50_ of artesunate and PSBMA‐ARTs conjugates against PHH cells was 413 and 500 µM, respectively. Figure S9 supports the result that the cytotoxicity of PSBMA‐ARTs conjugates was lower than that of HepG2 cells, although PSBMA‐ARTs conjugates could obviously be internalized into PHH cells. These results suggest that delivering PSBMA‐ARTs conjugates to cancer cells that rely on ferrous iron for survival can effectively induce cytotoxicity.

**FIGURE 7:**
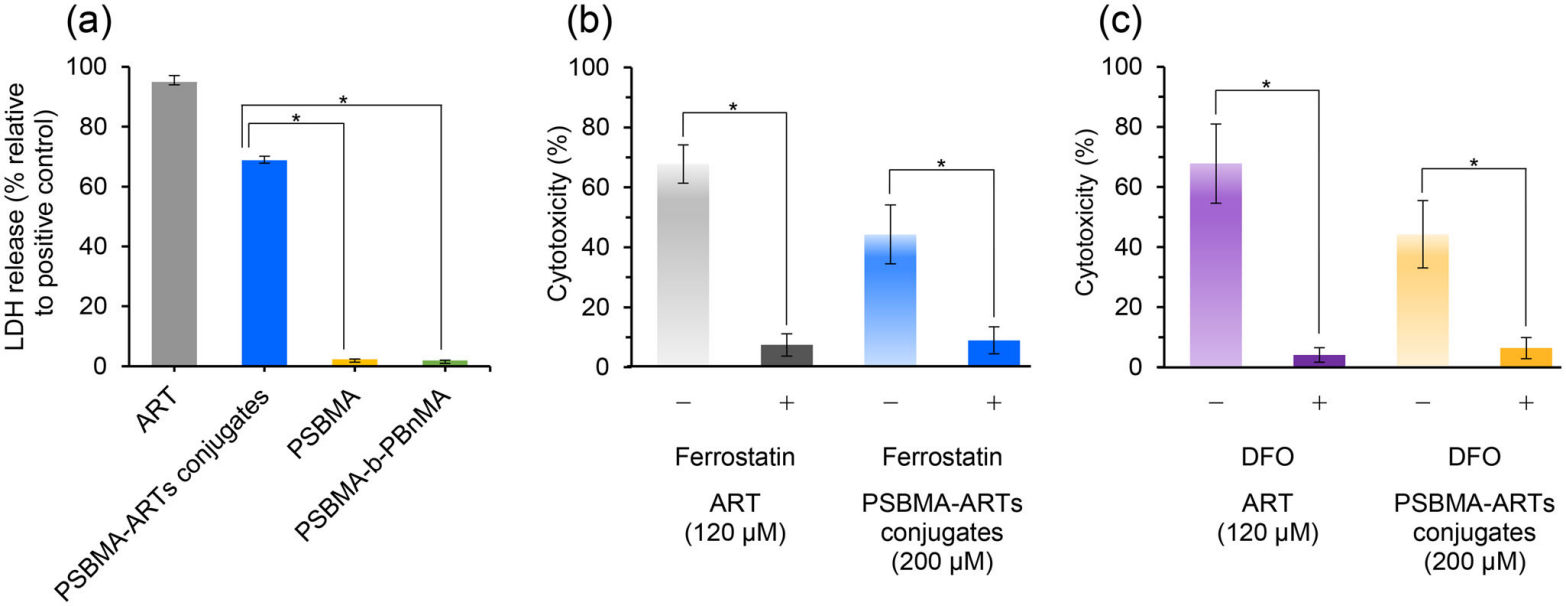
LDH assay of HepG2 treated with artesunate (ART), PSBMA‐ARTs conjugates, PSBMA, and PSBMA‐*b*‐PBnMA (a), and WST‐1 assay of HepG2 treated with ART or PSBMA‐ARTs conjugates after pretreatment with or without ferrostatin (b), deferoxamine (c). Both data are shown as the mean ± SD (*n* = 6). Statistical significance was measured between two groups using unequal variances Welch's t‐test. ^*^ *p* < 0.001.

The intracellular lactate dehydrogenase (LDH) would leak out from the cell due to the disruption of the cell membrane, inducing cell death. As shown in Figure 7a, the administration of artesunate at 120 µM and PSBMA‐ARTs conjugates at 200 µM to HepG2 cells, which was sufficient to induce cell death, resulted in cytotoxicity determined by LDH leakage from cells, similar to the WST‐1 assay. However, PSBMA without possessing poly(ART‐ethyl‐MA) segments, and PSBMA‐*b*‐PBnMA with a benzyl group introduced as the hydrophobic chain, showed almost no LDH leakage from HepG2 cells even at high concentrations (1,000 µg mL^−1^ as each polymer). Therefore, it is strongly suggested that PSBMA‐ARTs conjugates would generate free radicals through reaction with Fe^2^^+^ after internalization into cancer cells, and intracellular LDH might leak out due to the cell membrane disruption inducing ferroptosis.

To further investigate the involvement of ferroptosis in the cytotoxicity of PSBMA‐ARTs conjugates, the ferroptosis inhibition assays were performed using specific inhibitors. The allylic hydrogen of unsaturated fatty acids that constitute phospholipids in cell membranes and organelle membranes could readily be abstracted by free radicals, and carbon‐centered radicals would be formed [59]. Although the radicals generated would rapidly convert to peroxyl radicals by the addition of oxygen, the peroxyl radical may further abstract the hydrogen from water molecules or surrounding hydrocarbons to form hydroperoxides [60]. The generated hydroperoxides, whose ─O─O─ bond was thermodynamically weak, would be cleaved via Fenton reaction with Fe^2+^, and might generate extremely reactive alkoxy radicals that subsequently induce lipid peroxidation [61]. Ferrostatin‐1 (fer‐1) could act as a potent inhibitor of ferroptosis through the quenching of free radicals. It has been reported that free radical would be quenched by abstracting hydrogen from the secondary amine of fer‐1, and the generated aminyl radical would convert to nitroxyl radical via oxygen addition and its cleavage, and the nitroxyl radical could be nitroxide form by reducing free radicals [62]. As shown in Figure 7b, the cytotoxicity of HepG2 cells treated with artesunate or PSBMA‐ARTs conjugates was successfully canceled by fer‐1 administration, reducing it to less than 10%. Deferoxamine (DFO) is well known as a selective chelator of Fe^2+^. When HepG2 cells were exposed to artesunate or PSBMA‐ARTs conjugates after pretreatment with DFO, the cytotoxicity decreased to less than 10% (Figure 7c), similar to the results with fer‐1. These results demonstrated that the generation of free radicals from PSBMA‐ARTs conjugates would require Fe^2+^, and cancer cell death may be induced through the process of ferroptosis within HepG2 cells.

**FIGURE 8:**
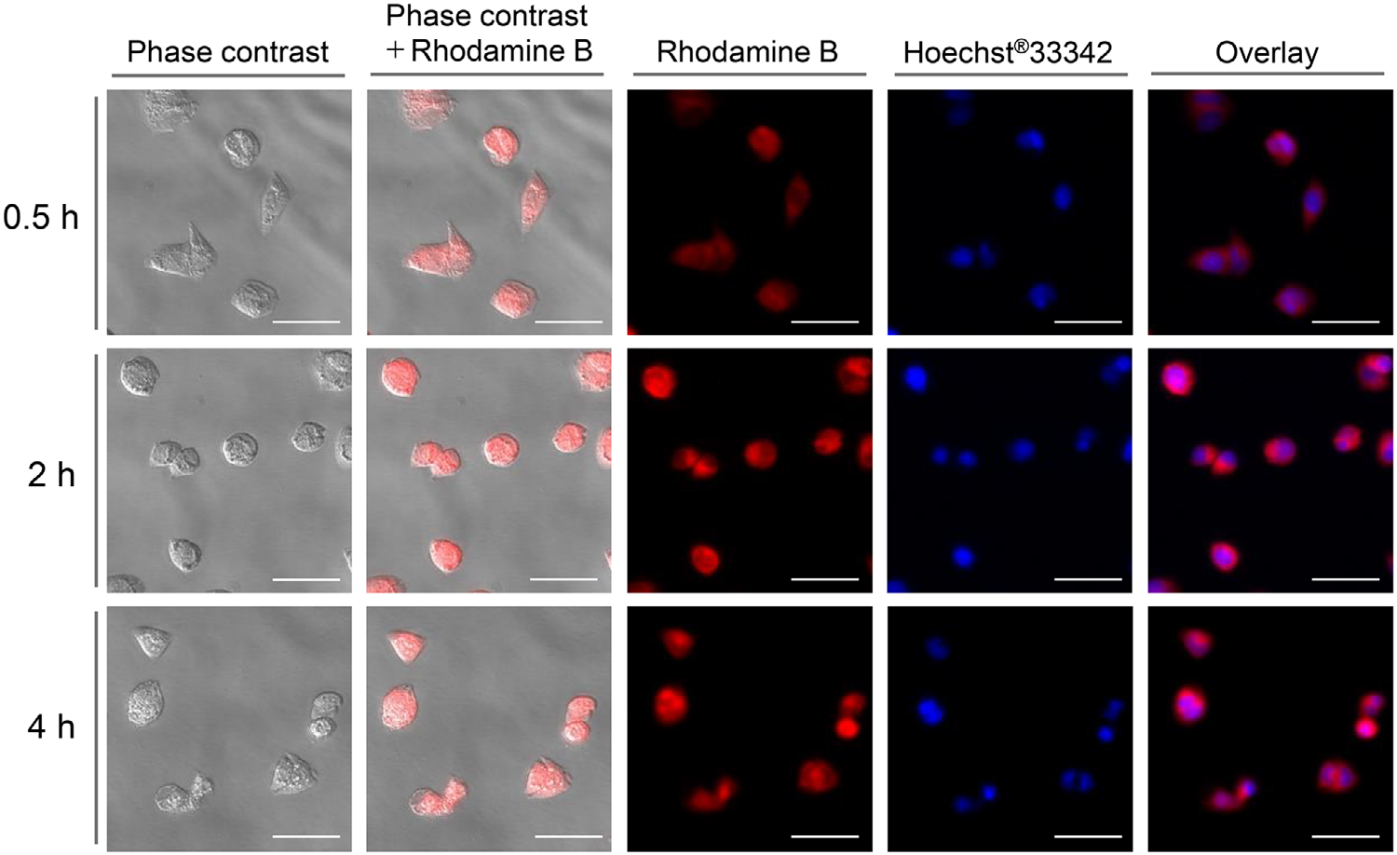
Cellular uptake of Rhodamine B‐labeled PSBMA‐ARTs conjugates. Scales bar = 50 µm.

Additionally, to demonstrate that the lipid peroxidation of HepG2 cells treated with PSBMA‐ARTs conjugates would be caused by the polymer internalized into the cells, the cellular uptake using Rhodamine B‐labeled PSBMA‐ARTs conjugates was investigated. As shown in Figure 8, the merged images of the phase contrast representing the cell morphology and the red fluorescence from polymer‐modified Rhodamine B revealed that the PSBMA‐ARTs conjugates could internalize into the cells after 30 min. The overlay between the blue fluorescence of the nucleus stained with Hoechst33342 and the red fluorescence of Rhodamine B indicated that PSBMA‐ARTs conjugates would diffuse into the cytoplasmic sol rather than into the nucleus.

**FIGURE 9:**
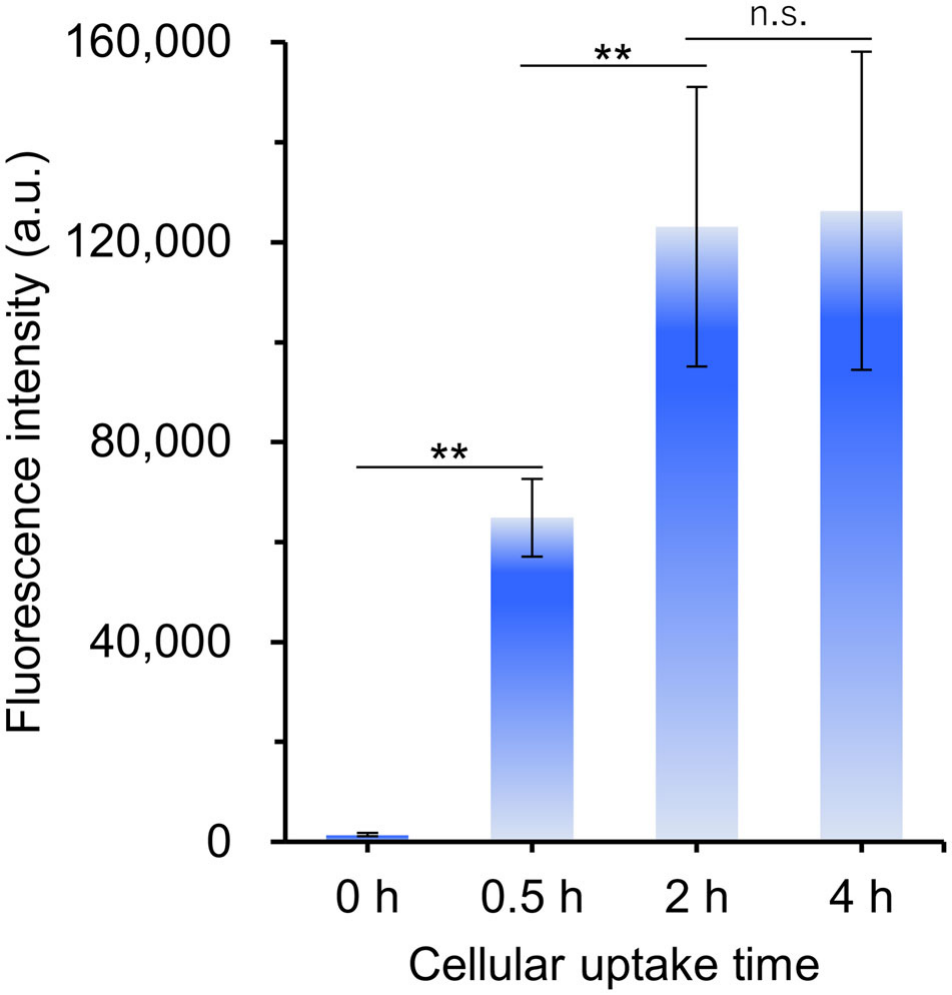
Brightness analysis of Rhodamine B‐labeled PSBMA‐ARTs conjugates internalized into HepG2 cells with time.

It can also be seen in Figure 9 that the internalization of PSBMA‐ARTs conjugates into HepG2 cells increased over 2 h and then became saturated, determined by the brightness analysis of the polymer migrated into the cells. These results ssuggest that PSBMA‐ARTs conjugates could internalize into HepG2 cells and subsequently generate free radicals via the reaction with ferrous ions in the cells, inducing the ferroptosis of HepG2 cells. Furthermore, to elucidate the internalization mechanism of PSBMA‐ARTs conjugates into cells, the cellular uptake assays were conducted using HepG2 cells pretreated with various endocytosis inhibitors. As shown in Figures S10 and S11, even in a 4°C environment that precludes ATP‐dependent endocytosis, in addition to pretreatment with inhibitors of caveolae‐ or clathrin‐mediated endocytosis and macropinocytosis, Rhodamine B‐labeled PSBMA‐ARTs conjugates could internalize into HepG2 cells as well as the blank at 37°C. Therefore, these results strongly suggest that PSBMA‐ARTs conjugates may migrate into HepG2 cells via ATP‐independent membrane permeation within a few hours. Since amphiphilic PSBMA incorporating PBnMA as a hydrophobic segments almost saturated the cellular uptake within 5 min in our previous study [30], the structure and incorporation rate of the hydrophobic segments introduced into PSBMA may be crucial parameters for water solubility and cell interaction.

### 3.5 CLSM Visualization of Lipid Peroxidation Induced by Free Radical Production From PSBMA‐ARTs Conjugates

Based on the above findings, PSBMA‐ARTs conjugates generated free radicals in a ferrous iron‐dependent manner and induced ferroptosis through their lipid peroxidation (LPO) activity. To demonstrate the intracellular LPO, the visualization of specific reactions between PSBMA‐ARTs conjugates and LPO may be necessary. It is well‐known that C11‐BODIPY is a useful imaging agent for detecting peroxidized lipids in cell‐ or intracellular membranes. C11‐BODIPY in the reduction state, which was retained in cell‐ or organelle membranes, would emit red fluorescence around 600 nm upon excitation by light of 560 nm. The olefinic moieties of C11‐BODIPY would partially be disrupted by the oxidation via the reaction with LPO, and the excitation light and fluorescence should undergo a blueshift (λ_max_ = 490 nm, λ_em_ = 530 nm). The LPO in HepG2 cells pretreated with artesunate alone or PSBMA‐ARTs conjugates at the same concentration as artesunate was evaluated by CLSM using C11‐BODIPY.

**FIGURE 10:**
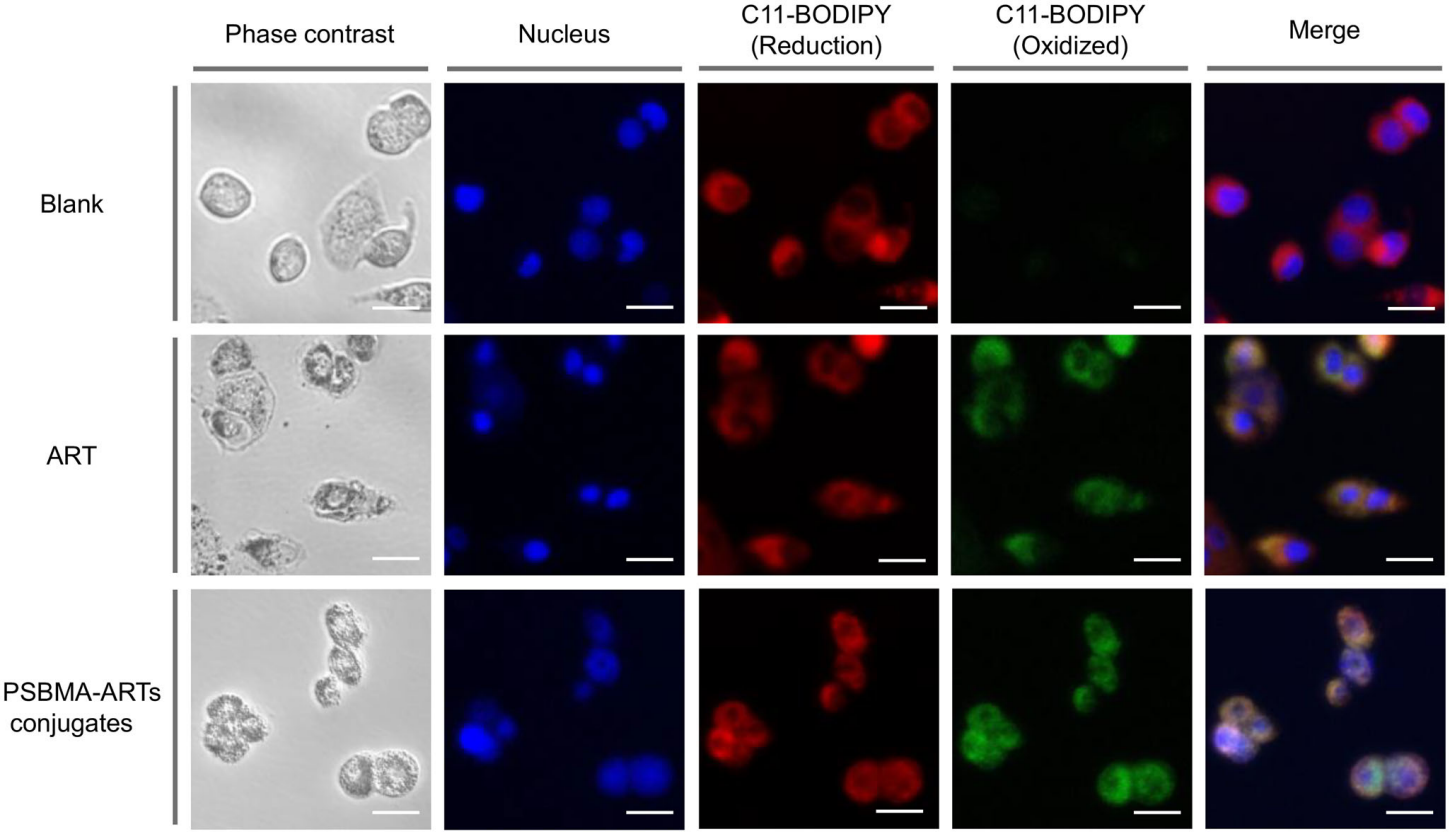
Intracellular LPO measurements with C11‐BODIPY 581/591 fluorescent probe in HepG2 cells as blank, pretreated with artesunate (ART) alone or PSBMA‐ARTs conjugates. Scales bar = 50 µm.

As shown in the upper column of Figure 10, the strong red fluorescence was observed in the blank cell excluding nuclei stained with Hoechst33342, whereas the green fluorescence generated by the reaction with LPO was significantly suppressed. It can also be seen in the middle and bottom columns of Figure 10 that the pretreatment of ART alone or PSBMA‐ARTs conjugates made red fluorescence of C11‐BODIPY decrease slightly compared to the blank, while green fluorescence within the cells increased. These results strongly support the occurrence of LPO within cells administered PSBMA‐ARTs conjugates, followed by ferroptosis.

**FIGURE 11:**
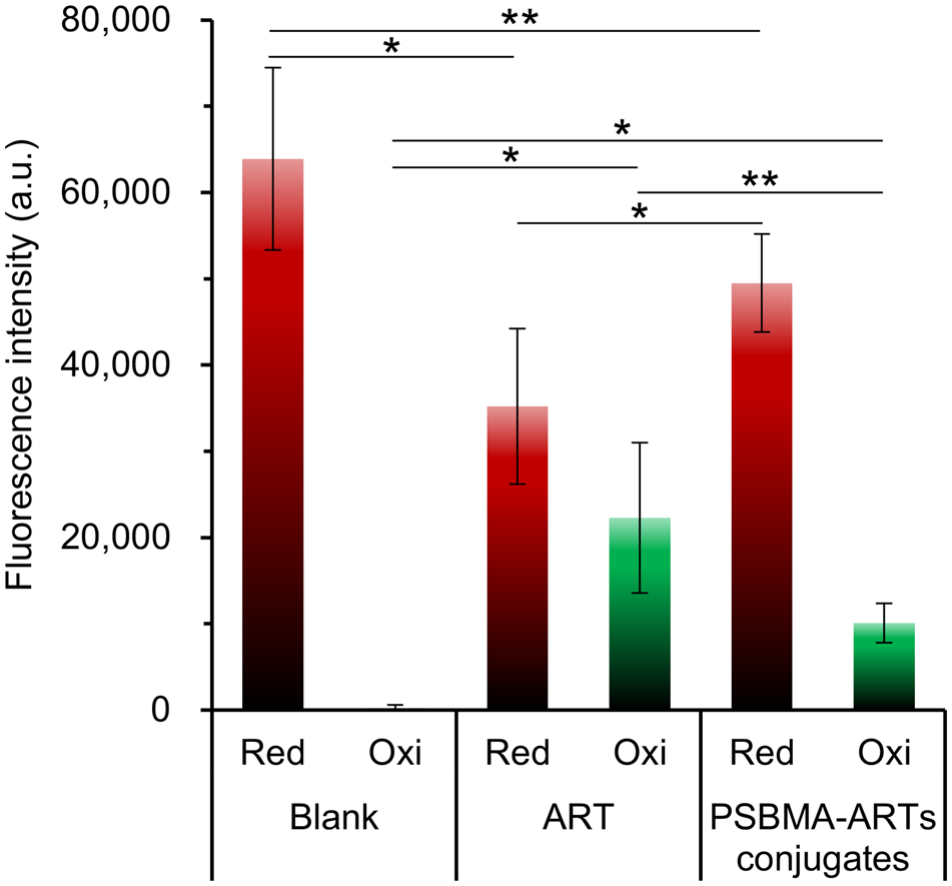
Brightness analysis of C11‐BODIPY reduced (Red) or oxidized (Oxi) in HepG2 cells, pretreated with or without artesunate (ART) alone or PSBMA‐ARTs conjugates. Both data are shown as the mean ± SD (*n* = 10). Statistical significance was measured between two groups using unequal variances Welch's t‐test. ^**^ *p* < 0.005, ^*^ *p* < 0.001.

In order to clarify the amount of LPO generated in cells, the brightness analysis was performed on the fluorescence intensities of C11‐BODIPY. As shown in Figure 11, the red fluorescence intensity (mean fluorescence intensity: 63,900) of C11‐BODIPY in the blank cell was significantly higher than the other, and LPO did not occur since the green fluorescence intensity (mean fluorescence intensity: 200) was markedly lower. ART exhibited a relative decrease in red fluorescence intensity (mean fluorescence intensity: 35,200), while the green fluorescence intensity (mean fluorescence intensity: 22,300) associated with LPO generation increased more than 20‐fold compared to the blank cell. The LPO induced by PSBMA‐ARTs conjugates (mean green fluorescence intensity: 10,100) was suppressed to approximately 50% of that induced by ART alone. The difference in LPO induction between them might be attributable to whether the drug administered directly to the cells was a small molecule or a macromolecule, and the low molecular weight ART would significantly exhibit higher diffusion rates and cellular uptake ratios compared to PSBMA‐ART conjugates. The utility of PSBMA‐ARTs conjugates may be evaluated through in vivo studies going forward.

## 4 Conclusion

Conjugating the artemisinin derivatives (ARTs) into the side chains of biocompatible polymers would be advantageous for effective cancer treatments through ferroptosis. The solid‐state copolymerization of SBMA and ARTs‐conjugated monomer proceeded to more than 91% conversion, and PSBMA‐ARTs conjugates were fabricated without any solvents or catalysts. The molecular weight and polydispersity of PSBMA‐ARTs conjugates were 8,000 g mol^−1^ and 1.10, respectively. PSBMA‐ARTs conjugates specifically produced free radicals in an aqueous solution containing ferrous ions. Since the particle size of PSBMA‐ARTs conjugates did not significantly increase in serum proteins dissolved in water, PSBMA‐ARTs conjugates would indicate the protein antifouling effects. The fluorescence‐labeled PSBMA‐ARTs conjugates could internalize into human hepatoblastoma cell lines (HepG2) via an ATP‐independent pathway. The cytotoxicity of iron‐responsive PSBMA‐ARTs conjugates caused ferroptosis of HepG2 cells, and the lipid peroxidation within cells has been visualized. Intriguingly, the IC_50_ (500 µM) of PSBMA‐ARTs conjugates for primary human hepatocytes was 2.37 times higher than that for HepG2 cells (211 µm), which may have high intracellular iron concentrations due to the high expression of transferrin receptors. These findings suggest that PSBMA‐ARTs conjugates would be promising as a ferroptosis inducer that does not require drug release, unlike conventional DDS carriers.

## Author Contributions

N. D. contributed to the conceptualization, methodology, validation, formal analysis, investigation, resources, data curation, visualization, funding acquisition, writing – original draft, and project administration. Y. Y. performed the methodology, validation, formal analysis, and investigation. Y. S. checked the methodology, validation, formal analysis, and investigation. R. N., A. K., M. I, T. Y., and S. Y. carried out the formal analysis and investigation. M. K. contributed to the conceptualization, methodology, and supervision. S. K. Supplied the conceptualization, methodology, resources, writing – review & editing, supervision, and project administration.

## Conflicts of Interest

The authors declare no conflicts of interest.

## Supporting information




**Supporting File**: marc70341‐sup‐0001‐SuppMat.docx.

## Data Availability

The data that supports the findings of this study are available in the supplementary material of this article.

## References

[1] Y. Matsumura and H. Maeda , “A New Concept for Macromolecular Therapeutics in Cancer Chemotherapy: Mechanism of Tumoritropic Accumulation of Proteins and the Antitumor Agent Smancs,” Cancer Research 46 (1986): 6387–6392, https://aacrjournals.org/cancerres/article/46/12_Part_1/6387/490212/A‐New‐Concept‐for‐Macromolecular‐Therapeutics‐in. 2946403

[2] H. Maeda , J. Wu , T. Sawa , Y. Matsumura , and K. Hori , “Tumor Vascular Permeability and the EPR Effect in Macromolecular Therapeutics: A Review,” Journal of Controlled Release 65 (2000): 271–284, 10.1016/S0168-3659(99)00248-5. 10699287

[3] H. Lai , T. Sasaki , N. P. Singh , and A. Messay , “Effects of Artemisinin‐Tagged Holotransferrin on Cancer Cells,” Life Sciences 76, no. 11 (2005): 1267–1279, 10.1016/j.lfs.2004.08.020. 15642597

[4] I. Nakase , H. Lai , N. P. Singh , and T. Sasaki , “Anticancer Properties of Artemisinin Derivatives and Their Targeted Delivery by Transferrin Conjugation,” International Journal of Pharmaceutics 354 (2008): 28–33, 10.1016/j.ijpharm.2007.09.003. 17942255

[5] I. Nakase , B. Gallis , T. Takatani‐Nakase , et al., “Transferrin Receptor‐Dependent Cytotoxicity of Artemisinin–Transferrin Conjugates on Prostate Cancer Cells and Induction of Apoptosis,” Cancer Letters 274 (2009): 290–298, 10.1016/j.canlet.2008.09.023. 19006645

[6] H. Lai , I. Nakase , E. Lacoste , N. P. Singth , and T. Sasaki , “Artemisinin‐Transferrin Conjugate Retards Growth of Breast Tumors in the Rat,” Anticancer Research 29 (2009): 3807–3810, https://ar.iiarjournals.org/content/29/10/3807. 19846912

[7] Y. Yang , X. Zhang , X. Wang , et al., “Enhanced Delivery of Artemisinin and Its Analogues to Cancer Cells by Their Adducts With Human Serum Transferrin,” International Journal of Pharmaceutics 467 (2014): 113–122, 10.1016/j.ijpharm.2014.03.044. 24661944

[8] H. Lai , T. Sasaki , and N. P. Singh , “Targeted Treatment of Cancer With Artemisinin and Artemisinin‐Tagged Iron‐Carrying Compounds,” Expert Opinion on Therapeutic Targets 9, no. 5 (2005): 995–1007, 10.1517/14728222.9.5.995. 16185154

[9] J.‐X. Dong , Z.‐D. Qi , Z.‐H. Zhang , and Y. Liu , “Standard Molar Enthalpies of Formation and Thermal Stabilities of Artemisinin and Its Two Derivatives: Artemether and Artesunate,” Journal of Chemical & Engineering Data 52 (2007): 1045–1049, 10.1021/je7000039.

[10] V. Galasso , B. Kovač , and A. Modelli , “A Theoretical and Experimental Study on The Molecular and Electronic Structures of Artemisinin and Related Drug Molecules,” Chemical Physics 335 (2007): 141–154, 10.1016/j.chemphys.2007.04.008.

[11] A. V. Pandey , B. L. Tekwani , R. L. Singh , and V. S. Chauhan , “Artemisinin, an Endoperoxide Antimalarial, Disrupts the Hemoglobin Catabolism and Heme Detoxification Systems in Malarial Parasite,” Journal of Biological Chemistry 274, no. 27 (1999): 19383–19388, 10.1074/jbc.274.27.19383. 10383451

[12] L. Nyadong , G. A. Harris , S. Balayssac , et al., “Combining Two‐Dimensional Diffusion‐Ordered Nuclear Magnetic Resonance Spectroscopy, Imaging Desorption Electrospray Ionization Mass Spectrometry, and Direct Analysis in Real‐Time Mass Spectrometry for the Integral Investigation of Counterfeit Pharmaceuticals,” Analitical Chemistry 81 (2009): 4803–4812, 10.1021/ac900384j. PMC4983440 19453162

[13] E. Ooko , M. E. M. Saeed , O. Kadioglu , et al., “Artemisinin Derivatives Induce Iron‐Dependent Cell Death (Ferroptosis) in Tumor Cells,” Phytomedicine 22, no. 11 (2015): 1045–1054, 10.1016/j.phymed.2015.08.002. 26407947

[14] G.‐Q. Chen , F. A. Benthani , J. Wu , D. Liang , Z.‐X. Bian , and X. Jiang , “Artemisinin Compounds Sensitize Cancer Cells to Ferroptosis by Regulating Iron Homeostasis,” Cell Death & Differentiation 27 (2020): 242–254, 10.1038/s41418-019-0352-3. 31114026 PMC7205875

[15] K. Batty , L. T. A. Thu , T. M. E. Davis , et al., “A Pharmacokinetic and Pharmacodynamic Study of Intravenous vs Oral Artesunate in Uncomplicated Falciparum Malaria,” British Journal of Clinical Pharmacology 45 (1998): 123–129, 10.1046/j.1365-2125.1998.00655.x. 9491824 PMC1873351

[16] P. N. Newton , Y. Suputtamongkol , P. T. Isavadharm , et al., “Antimalarial Bioavailability and Disposition of Artesunate in Acute Falciparum Malaria,” Antimicrobiology Agents and Chemotherapy 44 (2000): 972–977, 10.1128/aac.44.4.972-977.2000. PMC89800 10722499

[17] P. N. Newton , K. I. Barnes , P. J. Smith , et al., “The Pharmacokinetics of Intravenous Artesunate in Adults With Severe Falciparum Malaria,” European Journal of Clinical Pharmacology 62 (2006): 1003–1009, 10.1007/s00228-006-0203-2. 17089111

[18] G. Lee , J. S. Aneke , and D. J. Sullivan, Jr, “Fractional Curative Killing of Pyronaridine or Artesunate Combinations With Tafenoquine, 4‐Aminoquinolines, or Azithromycin in a Murine Malaria‐Luciferase Model,” Antimicrobiology Agents and Chemotherapy 69, no. 9 (2025): e00247–e00325, 10.1128/aac.00247-25. PMC12406666 40741950

[19] H. Nosrati , P. Barzegari , H. Danafar , and H. K. Manjili , “Biotin‐Functionalized Copolymeric PEG‐PCL Micelles for in vivo Tumor‐Targeted Delivery of Artemisinin,” Artificial Cells, Nanomedicine, and Biotechnology 47, no. 1 (2019): 104–114, 10.1080/21691401.2018.1543199. 30663422

[20] I. Leto , M. Coronnello , C. Righeschi , M. C. Bergonzi , E. Mini , and A. R. Bilia , “Enhanced Efficacy of Artemisinin Loaded in Transferrin‐Conjugated Liposomes Versus Stealth Liposomes Against HCT‐8 Colon Cancer Cells,” ChemMedChem 11, no. 16 (2016): 1745–1751, 10.1002/cmdc.201500586. 26999297

[21] H. K. Manjili , H. Malvandi , M. S. Mousavi , E. Attari , and H. Danafar , “In Vitro and in vivo Delivery of Artemisinin Loaded PCL–PEG–PCL Micelles and Its Pharmacokinetic Study,” Artificial Cells, Nanomedicine, and Biotechnology 46, no. 5 (2018): 926–936, 10.1080/21691401.2017.1347880. 28683649

[22] K. Liu , L. Dai , C. Li , J. Liu , L. Wang , and J. Lei , “Self‐Assembled Targeted Nanoparticles Based on Transferrin Modified Eight‐Arm‐Polyethylene Glycol–Dihydroartemisinin Conjugate,” Scientific Reports 6 (2016): 29461, 10.1038/srep29461. 27377918 PMC4932499

[23] L. Dai , L. Wang , L. Deng , et al., “Novel Multiarm Polyethylene Glycol‐Dihydroartemisinin Conjugates Enhancing Therapeutic Efficacy in Non‐Small‐Cell Lung Cancer,” Scientific Reports 4 (2014): 5871, 10.1038/srep05871. 25070490 PMC5376196

[24] N. Ito , A. Nabil , K. Uto , and M. Ebara , “Poly(ARTEMA), a Novel Artesunate‐Based Polymer Induces Ferroptosis in Breast Cancer Cells,” Science and Technology of Advanced Materials 26, no. 1 (2025): 2482514, 10.1080/14686996.2025.2482514. 40241849 PMC12001860

[25] N. Doi , Y. Yamauchi , R. Ikegami , M. Kuzuya , Y. Sasai , and S. Kondo , “Photo‐Responsive Polymer Micelles From *O*‐Nitrobenzyl Ester‐Based Amphiphilic Block Copolymers Synthesized by Mechanochemical Solid‐State Copolymerization,” Polymer Journal 52 (2020): 1375–1385, 10.1038/s41428-020-0387-9.

[26] M. Kuzuya , S. Kondo , A. Noguchi , and N. Noda , “Mechanistic Study on Mechanochemical Polymerization of Acrylamide,” Journal of Polymer Science, Part A: Polymer Chemistry 29 (1991): 489–494, 10.1002/pola.1991.080290405.

[27] S. Kondo , K. Murase , and M. Kuzuya , “Mechanochemical Solid‐State Polymerization. VI. Quantum Chemical Considerations for Structural Criteria of Mechanically Polymerizable Vinyl Monomers,” Chemical and Pharmaceutical Bulletin 42, no. 4 (1994): 768–773, 10.1248/cpb.42.768.

[28] S. Kondo , I. Hatakeyama , S. Hosaka , and M. Kuzuya , “Mechanochemical Solid‐State Polymerization (X): The Influence of Copolymer Structure in Copolymeric Prodrugs on the Nature of Drug Release,” Chemical and Pharmaceutical Bulletin 48, no. 12 (2000): 1882–1885, 10.1248/CPB.48.1882. 11145136

[29] N. Doi , Y. Yamauchi , Y. Sasai , K. Suzuki , M. Kuzuya , and S. Kondo , “Dextran‐Based Nanoparticles With 5‐FU‐Conjugated Polymethacrylate Segments for Drug Delivery: Synthesis of Amphiphilic Graft Copolymers by Mechanochemical Solid‐State Polymerization and Characterization,” International Journal of Biological Macromolecules 274 (2024): 132950, 10.1016/j.ijbiomac.2024.132950. 38848849

[30] N. Doi , Y. Yamauchi , Y. Sasai , et al., “Amphiphilic Sulfobetaine Copolymer for Boosting Internalization into Cancer Cells: Synthesis by Green Innovative Solid‐State Copolymerization and Characterization,” Macromolecules 58, no. 22 (2025): 12163–12180, 10.1021/acs.macromol.5c01571.

[31] N. Doi , Y. Yamauchi , Y. Sasai , et al., “Characterization of Mechanochemical Solid‐State Polymerization for Development of Sulfobetaine Polymer–Drug Conjugates: Design and Synthesis of Artemisinin‐Conjugated Novel Methacrylamide Derivatives,” Chemical and Pharmaceutical Bulletin 74, no. 3 (2026): 260–268, 10.1248/cpb.c26-00031. 41882826

[32] M. Kuzuya , S. Kondo , and K. Murase , “A Novel Single Electron Transfer in Solid‐State Organic Compounds: Mechanically Induced Reduction Of Dipyridinium Salts,” The Journal of Physical Chemistry 97, no. 30 (1993): 7800–7802, 10.1021/j100132a003.

[33] M. Kuzuya , S. Kondo , and A. Noguchi , “A New Development of Mechanochemical Solid‐state Polymerization of Vinyl Monomers: Prodrug Syntheses and Its Detailed Mechanistic Study,” Macromolecules 24, no. 14 (1991): 4047–4053, 10.1021/ma00014a013.

[34] T. Jin , M. Liu , K. Su , et al., “Polymer Zwitterion‐Based Artificial Interphase Layers for Stable Lithium Metal Anodes,” ACS Applied Materials & Interfaces 13, no. 48 (2021): 57489–57496, 10.1021/acsami.1c19479. 34839656

[35] P. M. O'Neill , A. Miller , L. P. D. Bishop , et al., “Synthesis, Antimalarial Activity, Biomimetic Iron(II) Chemistry, and in vivo Metabolism of Novel, Potent C‐10‐Phenoxy Derivatives of Dihydroartemisinin,” Journal of Medicinal Chemistry 44, no. 1 (2001): 58–68, 10.1021/jm000987f. 11141088

[36] A. Alberti , D. Macciantelli , and G. Marconi , “Free Radicals Formed by Addition of Antimalaric Artemisinin (Qinghaosu, QHS) to Human Serum: An ESR‐Spin Trapping Investigation,” Research on Chemical Intermediates 30, no. 6 (2004): 615–625, 10.1163/1568567041570366.

[37] M. Asano and H. Iwahashi , “Determination of the Structures of Radicals Formed in the Reaction of Antimalarial Drug Artemisinin With Ferrous Ions,” European Journal of Medicinal Chemistry 127 (2017): 740–747, 10.1016/j.ejmech.2016.10.053. 27823889

[38] A. R. Butler , B. C. Gilbert , P. Hulme , L. R. IRVINE , L. Renton , and A. C. Whitwood , “EPR Evidence for the Involvement of Free Radicals in the Iron‐Catalysed Decomposition of Qinghaosu (Artemisinin) and Some Derivatives; Antimalarial Action of Some Polycyclic Endoperoxides,” Free Radical Research 28, no. 5 (1998): 471–476, 10.3109/10715769809066884. 9702527

[39] L. L. Dugan , T.‐S. Lin , Y.‐Y. He , C. Y. Hsu , and D. W. Choi , “Detection of Free Radicals by Microdialysis/Spin Trapping Epr Following Focal Cerebral Ischemia‐Reperfusion and a Cautionary Note on the Stability of 5,5‐Dimethyl‐1‐Pyrroline N‐Oxide (DMPO),” Free Radical Research 23, no. 1 (1995): 27–32, 10.3109/10715769509064016. 7647917

[40] S. R. Meshnick , Y.‐Z. Yang , V. Lima , F. Kuypers , S. Kamchonwongpaisan , and Y. Yuthavong , “Iron‐Dependent Free Radical Generation from the Antimalarial Agent Artemisinin (Qinghaosu),” Antimicrobial Agents and Chemotherapy 37, no. 5 (1993): 1108–1114, 10.1128/AAC.37.5.1108. 8517699 PMC187911

[41] P. M. O'Neill , L. P. D. Bishop , N. L. Searle , et al., “Biomimetic Fe(II)‐Mediated Degradation of Arteflene (Ro‐42‐1611). The First EPR Spin‐Trapping Evidence for the Previously Postulated Secondary Carbon‐Centered Cyclohexyl Radical,” Journal of Organic Chemistry 65, no. 5 (2000): 1578–1582, 10.1021/jo991585m. 10814129

[42] W.‐M. Wu , Y. Wu , Y.‐L. Wu , et al., “Unified Mechanistic Framework for the Fe(II)‐Induced Cleavage of Qinghaosu and Derivatives/Analogues. The First Spin‐Trapping Evidence for the Previously Postulated Secondary C‐4 Radical,” Journal of the American Chemical Society 120, no. 14 (1998): 3316–3325, 10.1021/ja973080o.

[43] K. E. B. Doncom , H. Willcock , and R. K. O'Reilly , “The Direct Synthesis of Sulfobetaine‐Containing Amphiphilic Block Copolymers and Their Self‐Assembly Behavior,” European Polymer Journal 87 (2017): 497–507, 10.1016/j.eurpolymj.2016.09.002.

[44] Y. Wang and K. Matyjaszewski , “Catalytic Halogen Exchange in Miniemulsion ARGET ATRP: A Pathway to Well‐Controlled Block Copolymers,” Macromolecular Rapid Communications 41, no. 14 (2020): 2000264, 10.1002/marc.202000264. 32529731

[45] J.‐T. Wang , L. Wang , X. Ji , L. Liu , and H. Zhao , “Synthesis of Zwitterionic Diblock Copolymers with Cleavable Biotin Groups at the Junction Points and Fabrication of Bioconjugates by Biotin−Streptavidin Coupling,” Macromolecules 50, no. 6 (2017): 2284–2295, 10.1021/acs.macromol.6b02665.

[46] Q. Zhang , X. Tang , T. Wang , F. Yu , W. Guo , and M. Pei , “Thermo‐Sensitive Zwitterionic Block Copolymers via ATRP,” RSC Advances 4 (2014): 24240–24247, 10.1039/C4RA00971A.

[47] L. Li , Y. Wang , F. Ji , et al., “Synthesis and Characterization of Dendritic Star‐Shaped Zwitterionic Polymers as Novel Anticancer Drug Delivery Carriers,” Journal of Biomaterials Science, Polymer Edition 25, no. 14–15: Zwitterion Biomaterials (2014): 1641–1657, 10.1080/09205063.2014.936994. 25025700

[48] M. Tian , J. Wang , E. Zhang , J. Li , C. Duan , and F. Yao , “Synthesis of Agarose‐Graft‐Poly[3‐Dimethyl (Methacryloyloxyethyl) Ammonium Propanesulfonate] Zwitterionic Graft Copolymers via ATRP and Their Thermally‐Induced Aggregation Behavior in Aqueous Media,” Langmuir 29, no. 25 (2013): 8076–8085, 10.1021/la4007668. 23713658

[49] L.‐M. Zhang and L.‐Q. Chen , “Water‐Soluble Grafted Polysaccharides Containing Sulfobetaine Groups: Synthesis and Characterization of Graft Copolymers of Hydroxyethyl Cellulose With 3‐Dimethyl(Methacryloyloxyethyl)Ammonium Propane Sulfonate,” Journal of Applied Polymer Science 83, no. 13 (2002): 2755–2761, 10.1002/app.10191.

[50] T. Goda , K. Ishihara , and Y. Miyahara , “Critical Update on 2‐Methacryloyloxyethyl Phosphorylcholine (MPC) Polymer Science,” Journal of Applied Polymer Science 132, no. 16 (2015): 41766, 10.1002/app.41766.

[51] S. Yusa , K. Fukuda , T. Yamamoto , K. Ishihara , and Y. Morishima , “Synthesis of Well‐Defined Amphiphilic Block Copolymers Having Phospholipid Polymer Sequences as a Novel Biocompatible Polymer Micelle Reagent,” Biomacromolecules 6, no. 2 (2005): 663–670, 10.1021/bm0495553. 15762627

[52] K. Ishihara , “Revolutionary Advances in 2‐Methacryloyloxyethyl Phosphorylcholine Polymers as Biomaterials,” Journal of Biomedical Materials Research, part A 107, no. 5 (2019): 933–943, 10.1002/jbm.a.36635. 30701666

[53] T. Konno , K. Kurita , Y. Iwasaki , N. Nakabayashi , and K. Ishihara , “Preparation of Nanoparticles Composed With Bioinspired 2‐Methacryloyloxyethyl Phosphorylcholine Polymer,” Biomaterials 22, no. 13 (2001): 1883–1889, 10.1016/S0142-9612(00)00373-2. 11396894

[54] Z. Liu , S. Wang , W. Li , and Y. Tian , “Bioimaging and Biosensing of Ferrous Ion in Neurons and HepG2 Cells upon Oxidative Stress,” Analytical Chemistry 90, no. 4 (2018): 2816–2825, 10.1021/acs.analchem.7b04934. 29376642

[55] Y. Jiang , D. Hui , Z. Pan , et al., “Curcumin Promotes Ferroptosis in Hepatocellular Carcinoma via Upregulation of ACSL4,” Journal of Cancer Research and Clinical Oncology 150, no. 9 (2024): 429, 10.1007/s00432-024-05878-0. 39311951 PMC11420324

[56] Y. Li , Q. Ouyang , Z. Chen , et al., “Intracellular Labile Iron is a Key Regulator of Hepcidin Expression and Iron Metabolism,” Hepatology International 17, no. 3 (2023): 636–647, 10.1007/s12072-022-10452-2. 36512269

[57] A. T. Aron , M. O. Loehr , J. Bogena , and C. J. Chang , “An Endoperoxide Reactivity‐Based FRET Probe for Ratiometric Fluorescence Imaging of Labile Iron Pools in Living Cells,” Journal of the American Chemical Society 138, no. 43 (2016): 14338–14346, 10.1021/jacs.6b08016. 27768321 PMC5749882

[58] H.‐J. Chen , M. Sugiyama , F. Shimokawa , et al., “Response to Iron Overload in Cultured Hepatocytes,” Scientific Reports 10, no. 1 (2020): 21184, 10.1038/s41598-020-78026-6. 33273573 PMC7713074

[59] B. Maillard , K. U. Ingold , and J. C. Scaiano , “Rate Constants for the Reactions of Free Radicals With Oxygen in Solution,” Journal of the American Chemical Society 105, no. 15 (1983): 5095–5099, 10.1021/ja00353a039.

[60] H. Yin , L. Xu , and N. A. Porter , “Free Radical Lipid Peroxidation: Mechanisms and Analysis,” Chemical Reviews 111, no. 10 (2011): 5944–5972, 10.1021/cr200084z. 21861450

[61] G. Miotto , M. Rossetto , M. L. D. Paolo , et al., “Insight Into the Mechanism of Ferroptosis Inhibition by Ferrostatin‐1,” Redox Biology 28 (2020): 101328, 10.1016/j.redox.2019.101328. 31574461 PMC6812032

[62] O. Zilka , O. R. Shah , B. Li , et al., “On the Mechanism of Cytoprotection by Ferrostatin‐1 and Liproxstatin‐1 and the Role of Lipid Peroxidation in Ferroptotic Cell Death,” ACS Central Science 3, no. 3 (2017): 232–243, 10.1021/acscentsci.7b00028. 28386601 PMC5364454

