# Supplementary material for: Biocompatible Sulfobetaine Polymer‐Artemisinin Conjugates Inducing Ferroptosis in Cancer Cells: Synthesis by Mechanochemical Solid‐State Polymerization and Characterization
Source: Macromol Rapid Commun. 2026 Jun 22;47(15):e70341. doi: 10.1002/marc.70341 (PMC13435009; doi:10.1002/marc.70341)
Supplement: Supplementary file 1 [file MARC-47-e70341-s001.docx]

Supporting Information

Title

Biocompatible sulfobetaine polymer-artemisinin conjugates inducing ferroptosis in cancer cells: synthesis by mechanochemical solid-state polymerization and characterization

Naoki Doi,1 Yukinori Yamauchi,2 Yasushi Sasai,3 Ryosuke Nagai,1 Ayaka Koike1, Madoka Ikawa,1 Toru Yoshimoto,1 Satoshi Yamada,1 Masayuki Kuzuya,1† and Shin-ichi Kondo1*

Figure S1. ^1^H-NMR spectrum of SBMA.

Figure S2. ^1^H-NMR spectrum of APEMA.

Figure S3. ^1^H-NMR spectrum of artesunate.

Figure S4. ^1^H-NMR spectrum of ART-ethyl-MA.

Figure S5. ^1^H-NMR spectrum of purified (PSBMA)_21_-*b*-Poly(ART-ethyl-MA)_4_.

Figure S6. ^1^H-NMR spectrum of purified (PSBMA)_47_-*b*-Poly(ART-ethyl-MA)_8_.

Figure S7. Assumed scheme for the formation of radicals generated by the reaction of artemisinin or artesunate and ferrous ion.

Figure S8. HPLC chromatogram of artesunate aqueous solution as blank at 4 μg mL^-1^ (a), 2 μg mL^-1^ (b), 1 μg mL^-1^ (c), and calibration curve of peak area (d), and HPLC chromatogram of stability test of PSBMA-ARTs conjugates incubated for 12 h (e), 24 h (f), 48 h (g), 72 h (h), 96 h (i), 120 h (j), 144 h (k), and 148 h (l).

Figure S9. CLSM images of primary human hepatocytes treated with Rhodamine B-labeled PSBMA-ARTs conjugates for 30 min. Scales bar = 50 μm

Figure S10. CLSM images of HepG2 cells treated with Rhodamine B-labeled PSBMA-ARTs conjugates for 30 min after pretreatment with each endocytosis inhibitor. Scales bar = 50 μm

Figure S11. Brightness analysis of intracellular fluorescence intensity of Rhodamine B-labeled PSBMA-ARTs conjugates after pretreatment with each endocytosis inhibitor.
